# A Dry Powder Platform for Nose-to-Brain Delivery of Dexamethasone: Formulation Development and Nasal Deposition Studies

**DOI:** 10.3390/pharmaceutics13060795

**Published:** 2021-05-26

**Authors:** Laura Nižić Nodilo, Ivo Ugrina, Drago Špoljarić, Daniela Amidžić Klarić, Cvijeta Jakobušić Brala, Mirna Perkušić, Ivan Pepić, Jasmina Lovrić, Vesna Saršon, Maša Safundžić Kučuk, Dijana Zadravec, Livije Kalogjera, Anita Hafner

**Affiliations:** 1 Faculty of Pharmacy and Biochemistry, University of Zagreb, 10000 Zagreb, Croatia; lnizic@pharma.hr (L.N.N.); damidzic@pharma.hr (D.A.K.); cjakobus@pharma.hr (C.J.B.); mperkusic@pharma.hr (M.P.); ipepic@pharma.hr (I.P.); jlovric@pharma.hr (J.L.); 2 Faculty of Science, University of Split, 21000 Split, Croatia; iugrina@pmfst.hr; 3 Visage Technologies d.o.o., 10000 Zagreb, Croatia; drago.spoljaric@visagetechnologies.com; 4 Jadran-Galenski Laboratorij d.d., 51000 Rijeka, Croatia; vesna.sarson@jgl.hr (V.S.); masa.safundzic@jgl.hr (M.S.K.); 5 Department of Diagnostic and Interventional Radiology, University Hospital Center “Sestre milosrdnice”, University of Zagreb, 10000 Zagreb, Croatia; zadravec@sfzg.hr; 6 ORL/HNS Department, Zagreb School of Medicine, University Hospital Center “Sestre milosrdnice”, University of Zagreb, 10000 Zagreb, Croatia; kalogjera@sfzg.hr

**Keywords:** dexamethasone sodium phosphate, nose-to-brain delivery, spray-dried microspheres, pectin, hypromellose, mannitol, 3D nasal cavity model, in vitro nasal deposition

## Abstract

Nasal route of administration offers a unique opportunity of brain targeted drug delivery via olfactory and trigeminal pathway, providing effective CNS concentrations at lower doses and lower risk for adverse reactions compared to systemic drug administration. Therefore, it has been recently proposed as a route of choice for glucocorticoids to control neuroinflammation processes in patients with severe Covid-19. However, appropriate delivery systems tailored to enhance their efficacy yet need to emerge. In this work we present the development of sprayable brain targeting powder delivery platform of dexamethasone sodium phosphate (DSP). DSP-loaded microspheres, optimised employing Quality-by-Design approach, were blended with soluble inert carriers (mannitol or lactose monohydrate). Powder blends were characterized in terms of homogeneity, flow properties, sprayability, in vitro biocompatibility, permeability and mucoadhesion. Nasal deposition studies were performed using 3D printed nasal cavity model. Mannitol provided better powder blend flow properties compared to lactose. Microspheres blended with mannitol retained or enlarged their mucoadhesive properties and enhanced DSP permeability across epithelial model barrier. DSP dose fraction deposited in the olfactory region reached 17.0% revealing the potential of developed powder platform for targeted olfactory delivery. The observed impact of nasal cavity asymmetry highlighted the importance of individual approach when aiming olfactory region.

## 1. Introduction

The nasal route of administration offers a variety of therapeutic opportunities, including local-, systemic- and brain-targeted drug delivery. Currently it is attracting increasing scientists’ attention as a promising route of drug delivery in Covid-19 patients [1,2], particularly in severe cases presenting with central nervous system manifestations [3]. In particular nasally delivered drugs can easily access the CNS via the olfactory and trigeminal nerves bypassing the blood-brain barrier, thus providing effective CNS concentrations at lower doses and at lower risk for adverse reactions compared to systemic drug administration [4]. 

Nasal delivery of glucocorticoids has been proposed as an effective strategy to control neuroinflammation processes in patients with severe Covid-19, avoiding problems related to systemic application of high glucocorticoid doses [5]. Previously it was shown that nasally applied dexamethasone resulted in more efficient control of lipopolysaccharide-induced murine neuroinflammation when compared to intravenous administration of dexamethasone at the same dose [6]. Furthermore, nasal delivery of corticosteroids targeting olfactory region can be considered in the treatment of anosmia and hyposmia, frequently seen in Covid-19 patients [7]. 

The extent of direct nose-to-brain drug delivery greatly depends on the properties of drug delivery system and ability to reach targeted regions of nasal cavity [8]. Nasal powders offer important advantages over liquid formulations, including increased stability without the use of preservatives, prolonged residence time and higher drug concentration at nasal mucosa [9,10]. Powder formulations were previously shown as promising platforms for nose-to-brain drug delivery, as evidenced by animal studies [11,12,13,14,15]. However, the potential for nose-to-brain delivery in man cannot be easily drawn from data obtained in animal models due to discrepancy in their anatomical features [4]. 

In this work we present the development of nasal powder delivery platform for dexamethasone sodium phosphate (DSP), including consideration of nasal deposition in the early phase of formulation development. To our best knowledge, up to now there were no studies related to development of delivery system intended for nose-to-brain delivery of dexamethasone or its derivatives.

DSP is an ester prodrug of dexamethasone, freely soluble in water. When formulated in the dry powder form, it is expected to rapidly dissolve in contact with nasal fluid and to provide high drug concentration on the surface of the nasal epithelium, favouring drug absorption. On the other hand, due to its anionic nature, DSP is less permeable than dexamethasone base. Generally, for drugs with high solubility and low permeability, the rate limiting step in absorption process is the permeation across the nasal epithelium and in such cases, increase in nasal retention time is expected to augment the absorption the most [10]. Furthermore, absorption of phosphate ester prodrugs is promoted by their conversion to more permeable form by phosphatases that are present in human olfactory and respiratory nasal mucosa [16,17,18]. Antunes Viegas and co-workers demonstrated bioconversion of phosphate ester fosphenytoin to more permeable phenytoin by phosphatases in nasal porcine mucosa and nasal mucus from healthy human volunteers. In addition, they demonstrated nasal permeation potential of fosphenytoin itself. Recent study on fosphenytoin administration in mice proved the applicability of phosphate esters as useful strategy in nose-to-brain delivery of poorly soluble drugs [17,19]. 

Herein we propose a powder system consisting of spray-dried DSP-loaded pectin/hypromellose microspheres blended with mannitol or lactose monohydrate as inert carrier. Blending of microspheres with an inert carrier represents effective strategy to improve powder applicability. In optimised systems, drug-loaded microspheres as fine particles adhere to coarse carrier particles resulting in highly homogenous blend with improved dispersibility and nasal deposition profile [20,21]. 

A microsphere matrix composed of mucoadhesive polymers hypromellose and pectin is aimed to provide optimal swelling and drug release in contact with nasal fluid, ensuring prolonged retention at the deposition site and adequate rate and extent of drug delivery [22]. Furthermore, cellulose powder is well known gel forming (i.e., mechanical barrier forming) inert substance that is being used for the management and prevention of allergic rhinitis protecting mucosa from contact with allergens [23,24,25]. In that sense, it is reasonable to expect that the same concept might be applied to prevent the contact with airborne viruses, lowering the chance for viral respiratory co-infections.

In this study DSP-loaded microspheres were prepared by a spray drying method. The quality-by-design concept was applied to optimise the formulation (DSP and hypromellose concentration in the spray-drying feed) and process parameters (inlet air temperature and feed flow rate) in relation to microspheres’ physico-chemical properties, including process yield, DSP entrapment efficiency, residual moisture content, microsphere size and swelling properties. Optimised microspheres blended with inert carrier at different ratios were further characterised in terms of powder blend homogeneity, sprayability/flow properties, behaviour in contact with simulated nasal fluid, biocompatibility and DSP permeability across in vitro model of respiratory epithelial barrier. Nasal deposition pattern of the powder blend at various administration parameters was studied using 3D printed nasal cavity model, complementing the screening of its potential to ensure desired therapeutic effect.

## 2. Materials and Methods

### 2.1. Materials

DSP was purchased from Carbosynth Ltd. (Compton, UK). Low methoxy amidated pectin (CF 005, degree of esterification 35%; degree of amidation 15%; further denoted as pectin) was kindly donated by Herbstreith & Fox (Neuenbürg, Germany). Hypromellose (Metolose^®^ SH 4000) was obtained by courtesy of Shin-Etsu Chemical Co., Ltd., Tokyo, Japan. Lactose monohydrate (GranuLac^®^ 200; further denoted as lactose) was obtained from Meggle (Wasserburg am Inn, Germany). Mannitol was purchased from BDH Prolabo (Lutterworth, UK). Simulated Nasal Fluid (SNF) was prepared by dissolving NaCl (150.0 mM; Kemig, Zagreb, Croatia), KCl (40.0 mM; Kemig) and CaCl_2_ × H_2_O (5.3 mM; Sigma-Aldrich, Munich, Germany) in distilled water.

For cell biocompatibility and permeability studies in vitro, Hank’s balanced salt solution with 5.3 mM Ca^2+^ (HBSS-Ca^2+^; pH 7.4) was prepared by dissolving KCl (5.4 mM), NaHCO_3_ (4.2 mM), NaCl (136.9 mM), D-glucose monohydrate (5.6 mM) (all purchased from Kemig), KH_2_PO_4_ (0.4 mM; Kemika, Zagreb, Croatia), Na_2_HPO_4_ × 2H_2_O (0.3 mM; Fluka Chemie AG, Buchs, Switzerland) and CaCl_2_ × 2H_2_O (5.3 mM) (Sigma-Aldrich) in distilled water.

### 2.2. Statistical Design of Experiments

In order to optimise the formulation and process parameters for effective preparation of DSP-loaded microspheres suitable for nasal delivery, quality by design (QbD) principles were employed. The influence of four parameters at three levels was studied with a custom D-optimal experimental design that included varying formulation parameters such as the concentration of DSP and hypromellose and process parameters such as inlet air temperature and feed flow rate (Supplementary Materials; Table S1). To properly define design space i.e., lower and upper limits for parameter intervals from which corresponding parameter values in design will be chosen, preliminary experiments were performed. Spray drying process yield, drug loading (DL), entrapment efficiency (EE), particle size distribution, moisture content (MC) and swelling properties were investigated as responses. The analyses related to the responses were performed in triplicates, except for MC which was measured in duplicate. Design of experiments and data analyses were performed with the statistical software JMP 14.0 (JMP^®^, Version 14.0, SAS Institute Inc., Cary, NC, USA, © 1989–2007).

### 2.3. Preparation of Microspheres

DSP-loaded microspheres were prepared by spray drying of aqueous solutions of DSP and polymers—pectin and hypromellose. Firstly, concentrated aqueous solution of pectin (1.4%, *w*/*w*) and another of hypromellose (1.2%, *w*/*w*) were prepared. Pectin was dissolved at room temperature under stirring conditions for 24 h. Hypromellose solution was obtained after heating the water at 80–90 °C, followed by 24-h stirring at room temperature and storage in the refrigerator afterwards. DSP was dissolved in distilled water at concentrations of 1.0 and 10.0% (*w*/*w*). Spray-drying feed sample was prepared by mixing pectin and hypromellose solutions in suitable ratios, followed by addition of DSP solution, and distilled water when needed. Concentrations of polymers and DSP in feed solutions are shown in Table 1. 

DSP-loaded microspheres were obtained by spray-drying using a Büchi Mini Spray Dryer B–190 (Büchi, Flawil, Switzerland) equipped with a standard 0.7 mm nozzle. Two of the process parameters (aspirator capacity and compressed airflow) were constant, while inlet air temperature and feed flow rate varied between 120 and 160 °C and 2.5 and 4.5 g min^−1^, respectively. The process yield of each experiment was calculated as the ratio between the weight of obtained microspheres and the weight of DSP and polymers used for the preparation of the solution to be spray-dried.

### 2.4. Determination of Entrapment Efficiency and DSP Loading

The amount of DSP entrapped within the spray-dried microspheres was determined by the high performance liquid chromatography (HPLC) method as described in the Section 2.20. Microspheres (20 mg) were dispersed in distilled water and stirred for 24 h. Dispersion was filtered (0.2 μm) and analysed for DSP content. Each formulation was analysed in triplicate. Entrapment efficiency (EE) was determined as follows (Equation (1)):

EE (%) = *Q*_a_/*Q*_t_ × 100 (1)

where *Q*_a_ is the actual drug content in the examined amount of microspheres and *Q*_t_ is theoretical drug content in the examined amount of microspheres.

Drug loading (DL) was calculated using the following equation (Equation (2)):

DL (%) = *Q*_a_/*Q*_m_ × 100 (2)

where *Q*_m_ is the examined amount of microspheres.

### 2.5. Particle Size Distribution Measurement

Particle size distribution was determined by laser diffraction using a Malvern Mastersizer 3000 (Malvern Instruments Ltd., Malvern, UK), with a focal length of 300 mm, equipped with Hydro MV sample cell equipped with a magnetic stirrer. Approximately 5 mg of microspheres were dispersed in about 10 mL of ethanol (96%) by sonication in an ultrasonic bath until the suspension was homogeneous. Prior to the measurement, a background reading was made and the microsphere suspension was added to the Hydro MV cell until a 10–30% obscuration was reached. Before starting the measurement, the samples were equilibrated for 10 s. Each sample was analysed in triplicate. The results were expressed as volume diameters *D*v10, *D*v50 and *D*v90 and volume-weighted mean diameter *D*[4,3].

### 2.6. Moisture Content Determination

The moisture content in microspheres was analysed by thermogravimetric analysis using TGA Q500 (TA Instruments, New Castle, DE, USA). A small amount of microspheres was heated under dynamic nitrogen atmosphere of 25 mL min^−1^ to 150 °C at a rate of 10 °C min^−1^. Each sample was tested in duplicate. Moisture content was calculated using the following equation (Equation (3)):

(3) $$\mathrm{MC}\;\left(\%\right)=\frac{m_{0}-m_{e}}{m_{0}}\times 100$$

where *m*_0_ and *m_e_* are the weights of the powder at the beginning and at the end of experiment, respectively.

### 2.7. Swelling Study

Swelling properties of microspheres were analysed by an indirect method previously developed by our research group that includes using a Franz diffusion cell [22,26]. Microspheres were weighed (10 mg) directly on the polyamide membrane with 0.45 μm pore size (Sartorius Stedim Biotech GmbH, Goettingen, Germany), covering evenly the membrane surface. The receiver compartment was filled up with SNF or distilled water thermostated at 34 °C. At predetermined time intervals (3, 6, 9, 12 and 15 min), the receiver compartment was filled with fresh medium up to the initial level that lowered due to SNF/water uptake of the powder samples, using graduated microliter syringe (Hamilton, Bonaduz, Switzerland). Swelling properties of formulations were expressed as a volume of the medium uptaken by milligram of the microspheres. Three replicate measurements were performed for each sample.

### 2.8. Zeta-Potential Analysis

Zeta-potential of the microspheres was determined using Zetasizer Nano ZSP (Malvern Instruments) upon dispersion in 10 mM NaCl. All measurements were carried out at 25 °C. Each sample was analysed in triplicate.

### 2.9. *In Vitro* Release Study

In vitro DSP release study was performed using a Franz diffusion cell apparatus (25 mm: 20 mL volume, PermeGear, Hellertown, PA, USA) in which the receiver compartment was filled up with SNF and thermostated at 34 °C. In preliminary experiments, DSP was proven to be stable in SNF, at both 25 °C and 34 °C for 24 h. Sink conditions were provided during the whole experiment. The receiving medium was magnetically stirred during the whole experiment. The amount of (microspheres) powder sample containing 1.5 mg of DSP was weighed on the polyamide membrane (0.45 μm pore size) inserted between the donor and the receiver compartment. At scheduled time intervals, the samples (0.5 mL) were withdrawn from the receiver compartment and replaced with fresh medium in the total duration of 3 h. DSP content in the taken samples was determined by HPLC method as described in the Section 2.20. Each experiment was performed in triplicate.

### 2.10. Preparation of Powder Blends

Lactose and mannitol powders with particle size ranging between 45 and 63 μm were used as carriers. This powder fraction was separated using a laboratory sieve shaker (Vibratory Sieve Shaker AS 200, Retsch, Haan, Germany) equipped with 20 cm diameter sieves (Retsch^®^; nominal aperture 63 and 45 μm). The system was vibrated for 10 min at an amplitude of 50%. Powder blends were prepared by mixing microsphere and carrier powder at 1:9 and 1:19 weight ratios. After premixing for 5 min using MX-S vortex mixer (1250 rpm; DLAB Scientific Co. Ltd., Beijing, China) in 50 mL centrifuge tube (Falcon^®^, Corning Costar Inc. Tewksbury, MA, USA), microspheres and carrier were transferred in 1.5 mL centrifuge tube (Eppendorf, Hamburg, Germany) and mixed using a Turbula^®^ shaker mixer (WAB Group, Muttenz, Switzerland) operating for 10 min at 70 rpm.

Homogeneity of microspheres/carrier blends was evaluated based on the analysis of drug content in the powder samples (10 mg) taken from the top, centre and bottom of the centrifuge tube. Powder samples were dissolved in 10.0 mL of distilled water. After filtering (pore size 0.2 µm), the solutions were analysed for DSP content by the HPLC method as described in the Section 2.20. All samples were taken in triplicate.

Adequate homogeneity was assumed for the powder blends that showed mean percentage ratio of experimentally determined and theoretical mass of DSP in the analysed samples within the range of 100.0 ± 5.5%, with a relative standard deviation (RSD) value ≤5.5% [27].

### 2.11. Scanning Electron Microscopy

The morphology and surface of the microspheres and microspheres/carrier powder blends was observed by scanning electron microscopy (SEM) using a VEGA3 microscope (TESCAN, Brno, Czech Republic) operating at an acceleration voltage of 20 kV. The powder samples were placed on a metal stub using a double-sided adhesive tape and were coated with a thin layer of gold and palladium applying a Quorum SC7620 sputter coater (Emitech, London, UK) under 0.01 mbar vacuum and inert argon atmosphere.

### 2.12. Powder Flow Properties

Powder flow properties were determined by indirect method by measuring tapped and bulk density of the powders. The experiment was performed according to Kaialy et al. [28] with slight modifications. Powder (200 mg) was weighed and filled in a 5 mL syringe and the volume was recorded. Then, the syringe was tapped until there was no change in the powder volume (around 100 times) and the new volume was recorded. Hausner ratio was calculated according to the formula (Equation (4)):

(4) $$Hausner\;ratio=\frac{Tapped\;density}{Bulk\;density}$$

### 2.13. Spray Cone Angle Determination

Spray cone angle was measured by a virtual protractor after spraying the powder samples against a dark background [29]. Powder samples (15 mg) were weighed in a hypromellose capsule (Vcaps^®^ Plus, size 3, kindly donated by Capsugel, Lonza, Basel, Switzerland). The capsule was placed in Miat^®^ monodose nasal insufflator (Miat S. p. A., Milan, Italy), pierced and the powder sample was actuated by squeezing a rubber bulb to generate airflow. The emitted plume was recorded using a camera Panasonic Lumix DMCFZ1000 (Panasonic, Osaka, Japan) of 120 frames per second and afterwards analysed for the spray cone angle. Each formulation was tested in triplicate.

### 2.14. *In Vitro* Mucoadhesion Test

Nasal mucosa was isolated from porcine heads obtained from a local abattoir following procedure described by Fachel et al. [30]. The head was split in two halves by longitudinal incision exposing septum and conchae in the nasal cavities. The mucosa was carefully separated from the underlying tissue and stored at −20 °C until use. Mucoadhesive properties of microspheres and their blends with lactose or mannitol were determined according to the slightly modified method of Jurišić Dukovski et al. [31] using TA.XT Plus Texture Analyser (Stable Micro Systems, Godalming, UK) equipped with the Mucoadhesion Rig. Briefly, nasal porcine mucosa was cut into circular sections with a 10 mm diameter, which were then fixed to the upper probe with a cyanoacrylate glue. The amount of 5 mg of powder samples was weighed onto the lower platform of the mucoadhesion rig and was moisturized with SNF (20 µL) for 30 s. The mucosal section on the upper probe was soaked in SNF thermostated at 34 °C for 30 s prior to measurement. The settings of the test were as follows: pre-test speed 0.5 mm s^−1^, test speed 0.1 mm s^−1^, contact time 30 s, applied force 0.1 N and post-test speed 0.1 mm s^−1^. All experiments were performed in triplicate. The maximum detachment force (*F*_max_) and the work of adhesion (*W*_adh_) were used as a measure of mucoadhesive properties. The work of adhesion was calculated according to the following equation (Equation (5)):

(5) $$W_{adh}=A\times 0.1\times 1000_{\;}$$

where *A* corresponds to the area under the force-distance plot, 0.1 represents the conversion of the time measurement to distance (the sample was raised at 0.1 mm s^−1^), and multiplication by 1000 serves to express the values in µJ.

### 2.15. Cell Culture Conditions

Calu-3 cell line (ATCC^®^ HTB-55^™^) was obtained from ATCC (Manassas, VA, USA). The cells were cultured in DMEM-F12 cell culture medium (Sigma Aldrich, St. Louis, MO, USA), supplemented with penicillin/streptomycin (1% *v*/*v*; Lonza) and foetal bovine serum (FBS; 10% *v*/*v;* Sigma Aldrich). The cell cultures were maintained in the incubator (Sanyo CO_2_, Nagasaki, Japan) at 37 °C, 5% CO_2_. The culture medium was changed every 2–3 days and the cells were passaged when 70–90% confluence was reached. The cells were detached from the flasks by trypsin/EDTA (0.25%/0.02% in phosphate-buffered saline, PBS, respectively). The cells were split in ratios from 1:3 to 1:6. The passages used for biocompatibility and permeation studies were 13–15 and 13, 19 and 23, respectively.

### 2.16. *In Vitro* Biocompatibility Studies

The cells were seeded in 96 well plates (Corning Costar, Corning, NY, USA) at density of 4 × 10^4^ cells per well and were used for biocompatibility studies after 48 h of culturing. Microspheres and microspheres/carrier powder blends were homogeneously dispersed in distilled water at DSP concentration ranging from 0.9 to 3.68 mg mL^−1^, and corresponding inert carrier concentration range from 25.4 to 214.4 mg mL^−1^. DSP, mannitol and lactose solutions at concentrations equal to those in powder sample dispersions were used as controls. The dispersions and solutions were mixed with HBSS-Ca^2+^ (pH 7.4) in volume ratio 1:1, reaching the final concentration in the range of 0.45–1.8 mg mL^−1^ for DSP and 12.7 to 107.2 mg mL^−1^ for lactose or mannitol per well. Before the treatment, cell culture medium was withdrawn, cells were washed with HBSS-Ca^2+^ and exposed to the prepared dispersions or solutions for 2 h at 37 °C. Cells incubated in HBSS-Ca^2+^ were used as a negative control. After the treatment, the cells were washed twice with HBSS-Ca^2+^ and the biocompatibility test was performed. Cell viability was determined by colorimetric MTT (3-[4,5-dimethylthiazol-2-yl]-2,5-diphenyl tetrazolium bromide, Sigma-Aldrich) assay. The reagent was prepared by dissolving MTT (2.5 mg mL^−1^) in PBS (Lonza), followed by the addition of DMEM-F12 cell medium to obtain the concentration of 0.5 mg mL^−1^. In each well 100 µL of the MTT solution was added, after which the cells were incubated for 30 min at 37 °C. Afterwards, the medium was removed, the cells were lysed and the formazan crystals dissolved by the addition of 100 µL of isopropanol per well. The amount of formazan was determined spectrophotometrically at 570 nm (1420 Multilabel counter VICTOR3, Perkin Elmer, Waltham, MA, USA). Metabolic activity was expressed as relative to control (untreated cells incubated in HBSS-Ca^2+^) according to the following equation (Equation (6)):

(6) $$\mathrm{Viability}\;\left(\%\right)=\frac{A_{sample}-A_{ipr}}{A_{c}-A_{ipr}}\times 100$$

where *A*_sample_ is the absorbance of a solution of formazan crystals formed in cells treated with tested samples, *A*_c_ is the absorbance of a solution of formazan crystals formed in untreated cells (exposed to HBSS-Ca^+^) and *A*_ipr_ is the absorbance of pure isopropanol.

### 2.17. *In Vitro* Permeability through Epithelial Model Barrier

In vitro permeability studies of DSP were performed using Calu-3 cell monolayer according to procedure described by Matilainen et al. [32]. Calu-3 epithelial cells were seeded onto the polycarbonate 12-well Transwell^®^ inserts (0.4 μm mean pore size, 1.12 cm^2^ surface area; Corning Costar Inc.) at a density of 5.5 × 10^5^ cells per well. The cells were cultured with the cell culture medium volume of 0.5 mL in the apical and 1.5 mL in the basolateral compartment. After 48 h, cell culture medium was removed from the apical compartment to create an air interface and the cells were grown with 800 μL of culture medium in the basolateral compartment. The cell culture medium in basolateral compartment was changed every other day and 24 h prior to the permeability experiment. The transepithelial electrical resistance (TEER) of the monolayers was measured using epithelial volt/ohm meter EVOM equipped with STX-2 chopstick electrode (WPI Inc., Sarasota, FL, USA) to determine the formation of the monolayers and their integrity. The cells were grown on Transwell^®^ membranes for 12–14 days, until the plateau of TEER values was reached (above 1000 Ω cm^2^). Permeability studies were carried out in HBSS-Ca^2+^. In order to determine permeation profile of DSP and dexamethasone base (DB) across Calu-3 monolayer from isoosmotic solution, DSP and DB were dissolved in HBSS-Ca^2+^ at concentration of 0.9 mg mL^−1^ and 0.075 mg mL^−1^, respectively. Other samples for permeability experiments were prepared by dissolving/dispersing the appropriate amount of DSP, DSP-MS and DSP-MS/carrier in distilled water obtaining DSP concentration of 1.8 mg mL^−1^. In addition, solutions of DB and DB/carrier in distilled water at DB concentration of 0.15 mg mL^−1^ were also prepared. Aqueous solutions/dispersions were then mixed with HBSS-Ca^2+^ in 1:1 ratio, reaching the final DSP concentration of 0.9 mg mL^−1^ or final DB concentration of 0.075 mg mL^−1^. Concentration of mannitol in the test samples containing mannitol was 54.5 mg mL^−1^. HBSS-Ca^2+^ was used as negative control for TEER values and cell viability. Osmolality of all test samples was measured using OsmoTECH^®^ Single-Sample Micro-Osmometer (Advanced Instruments, Norwood, MA, USA).

Prior to the experiment, the monolayers were washed with HBSS-Ca^2+^ and HBSS-Ca^2+^ was placed into the apical (0.5 mL) and basolateral (1.5 mL) compartments. The cell monolayers were then incubated for 20 min at 37 °C, 5% CO_2_. At the beginning of the experiment, the apical compartment was emptied and 0.5 mL of the tested sample or HBSS-Ca^2+^ was added. Samples (0.5 mL) were taken from the basolateral compartment at regular time intervals over 120 min and replaced with the same volume of fresh thermostated HBSS-Ca^2+^ (37 °C). During the permeability experiment, monolayers were incubated at 37 °C and 50 rpm on a horizontal orbital shaker. The TEER values were measured before, during and after the permeation experiments to check the cell layer integrity. At the end of the experiment, the samples from the apical compartments were also collected. All experiments were done in triplicate. Samples were analysed for drug content using HPLC (Section 2.20). The apparent permeability coefficient (*P*_app_) was calculated according to the following equation (Equation (7)):

(7) $$P\mathrm{app}=\frac{dQ}{dt}\times \frac{1}{AC_{0}}$$

where d*Q*/d*t* is the permeability rate, *A* is the surface area of the permeation barrier and *C*_0_ is the initial concentration of drug in the apical compartment [33].

The biocompatibility of the formulations by MTT assay was also examined on Calu-3 cell monolayer grown on Transwell^®^ plates 24 h after the permeability assay. A 5 μg mL^−1^ MTT (3-[4,5-dimethylthiazol-2-yl]-2,5-diphenyl tetrazolium bromide, Sigma-Aldrich) solution was prepared in PBS and diluted with DMEM-F12 cell medium to a final MTT concentration of 0.5 mg mL^−1^. MTT solution (0.7 mL) was added to both the basolateral and apical compartment. Treated cells were incubated for 30 min at 37 °C. After 30 min, the medium was removed and 0.7 mL of isopropanol was added to both the apical and basolateral compartment. The Transwell^®^ plate was placed on an orbital shaker at room temperature to facilitate the dissolution of the formed formazan crystals. The plate was covered with aluminium foil for protection from light. After dissolving formazan, solutions from the apical and basolateral compartments were combined and stirred, and 100 μL of the solution for each sample was transferred to a 96-well plate in triplicate. Absorbance was measured at a wavelength of 570 nm (Victor, PerkinElmer, Waltham, MA, USA). Cell viability was expressed as relative to control as described in the Section 2.15.

### 2.18. Development of Nasal Cavity Model

Multi-sectional nasal cavity model was developed based on anonymized Computed Tomography (CT) scan of a 62-year old patient, obtained from Sisters of Charity Hospital database. The patient had healthy nasal airway passages, as confirmed by a specialist. Reconstruction, design and 3D print of a nasal cavity model was performed by CATEH d.o.o., Zagreb, complying with ISO 13485 standards. InVesalius 3.1 software was used to segment CT and reconstruct the nasal cavity model while Rhinocheros^®^ 7 was used to design the nasal cavity model in multiple separate pieces. Nasal cavity model was fragmented into anterior region, turbinate region with detachable olfactory segment, septum with detachable olfactory segment and posterior region/nasopharynx equipped with a connector for respiratory pump used to produce adequate inspiration pattern. The model also includes paranasal sinuses with openings into the nasal cavity. Bar pins with 6.4 and 2.0 mm diameter and 6.0 and 4.0 mm height and transverse coupling were used to ensure proper assembly and alignment of the model segments.

The model was produced by stereolithography using 3D Systems^®^ProX 800 (3D Systems, Inc., Rock Hill, SC, USA). It was printed in transparent rigid plastic Accura ClearVue, with the resolution of 10 µm layer thickness. In order to enable appropriate nasal device insertion into the nostrils, anterior region was printed in flexible material (DigitalMaterial FLX 9850, 60 ShoreA TangoBlackPlus and VeroWhitePlus) at a resolution of 30 µm layer thickness using Stratasys Connex 350 printer (Stratasys Ltd, Rehovot, Israel).

### 2.19. Assessment of the Deposition Profile within the Nasal Cavity *In Vitro*

The nasal cavity model was placed on a stand and connected to Respiratory Pump Model 613 (Harvard Apparatus, Holliston, MA, USA), simulating breathing at inspiratory flow rate of 0 L min^−1^ (no breathing) or 20 L min^−1^ (representing moderately deep inspiration of the patient) [21]. Volumetric flow rate was checked by nasal inspiratory flow meter In-Check Nasal (Clement Clarke International Ltd., Harlow, UK). During inspiration with one nostril blocked, powder formulation was sprayed with Miat^®^ device to the free nostril of the nasal cavity model at a nostril insertion depth of 5 mm, at actuation angle 0° from the vertical plane, and 60 or 75° from the horizontal plane. Fractional spray deposition pattern was quantitatively determined after thorough rinsing of each segment of the nasal cavity with distilled water. Eluates were characterised in terms of drug content by the HPLC method described in the Section 2.20. Drug deposited in each region was expressed in relation to total dose. For determination of total dose recovery, the fraction of the dose retained in the capsule after device actuation was also evaluated. Three replicate assessments of deposition pattern were conducted for each formulation.

### 2.20. Quantitative Determination of DSP and DB

Quantitative determination of DSP and DB was performed by HPLC. The chromatographic system consisted of an autosampler, system controller, pump, degasser, an UV–VIS detector, a column oven, all Series 200 (PerkinElmer). TotalChrome Navigator software for data processing was used for all chromatographic analyses. Satisfactory separation was achieved on Kinetex C18 (100 × 4.6 mm, 2.6-μm particle size) reverse-phase column with suitable guard column, both obtained by Phenomenex (Torrance, CA, USA). The chromatographic conditions were a modification of those by Jurišić Dukovski et al. [31]. The mobile phase consisted of 68% 5 mM acetate buffer (pH 4.5) and 32% acetonitrile (*v*/*v*). The isocratic elution was carried out at a flow rate of 0.7 mL min^−1^ and the column was thermostatically controlled to a temperature of 55 °C. The injection volume was 20 μL. The detection wavelength for each analyte was at 241 nm. The corresponding concentrations were determined from the integrated peak area using the appropriate calibration curves. The proposed method was validated based on the International Conference on Harmonization (ICH) guideline Q2 (R1) [34]. Validation of the method was carried out for selectivity, linearity, range, accuracy, repeatability, intermediate precision, limit of detection (LOD) and limit of quantification (LOQ) (Table S2, Figure S1).

### 2.21. Statistical Analysis

Statistical data analyses were performed on all data using a one-way ANOVA followed by a Tukey’s post hoc test with *p* < 0.05 set as the minimal level of significance. Calculations were performed using JMP 14.0 software (JMP^®^, Version 14.0, SAS Institute Inc., Cary, NC, USA, 1989–2007).

To evaluate the correlation between two variables (i.e., percentage of the dose retained within the capsule vs. Hausner ratio and spray cone angle vs. Hausner ratio), Pearson’s (parametric) correlation coefficient was determined using the same statistical software. Significance level was set to 0.05.

## 3. Results and Discussion

This study focused on the development of dry powder platform for brain targeted dexamethasone delivery consisting of DSP-loaded pectin/hypromellose microspheres prepared by spray drying and mannitol or lactose as inert carrier.

DSP is a water-soluble sodium phosphate salt of dexamethasone, suitable for formulation procedures such as aqueous solution spray drying. Apart from enabling the administration of higher drug doses and achieving high drug bioavailability owing to permeation and/or conversion to diffusible active form in vivo, water-soluble phosphate ester prodrugs are easy to formulate with no need for potentially toxic excipients [19]. Pectin and hypromellose are biocompatible and mucoadhesive polymers already used as constituents in approved liquid nasal formulations, such as PecFent (approved by the European Medicines Agency) and Astelin (approved by The United States Food and Drug Administration, FDA). Hypromellose presented a polymer of choice in several studies aimed at development of nasal powders intended for CNS drug delivery [13,15]. Hypromellose showed mucoadhesive properties and provided appropriate drug release profile in relation to route of administration [15]. Our group previously demonstrated that combining hypromellose with pectin improved polymer matrix properties ensuring moderate swelling in contact with nasal fluid, due to pectin crosslinking in the presence of calcium ions [22]. For each API to be incorporated in pectin/hypromellose matrix it is important to optimise pectin to hypromellose and API to polymers weight ratios in the spray dried solution.

Both selected inert carriers (lactose and mannitol) are listed in the FDA database of inactive ingredients for nasal or respiratory use [35]. Pharmaceuticals and Medicals Devices Agency of Japan approved nasal powder formulation of dexamethasone cipecilate as dexamethasone ester prodrug for the treatment of allergic rhinitis (Erizas^®^), comprising active pharmaceutical ingredient and lactose as an additive. Mannitol is a chemically inert highly water-soluble pharmaceutical excipient that has been considered as a bulking (matrix forming) agent and/or carrier in development of various nasal powders such as vaccine formulations [36], systemic [37] and nose-to-brain drug delivery platforms [11]. Mannitol has been demonstrated to alter mucosal permeability by osmotic effect [38,39]. In addition, lactose and mannitol are expected to provide beneficial effect on the polymer network hydration status of the swollen microspheres. Namely, the moisturising effect of carriers is known to prevent drying out of the gel and formation of the polymeric crusty layer that might irritate mucosa [40].

### 3.1. Experimental Design: Analysis of the Results

Within this work, experimental design was employed for fine tuning of the formulation and process parameters with the aim to optimise microspheres’ physicochemical properties.

Drug/polymers solution spray drying following the design matrix (Table 1) resulted in dry powder product for every experimental run approving the appropriateness of the formulation and process parameter settings within the design of experiment.

Microspheres prepared were characterised in terms of drug entrapment efficiency, drug loading, particle size and particle size distribution, residual moisture content and swelling ability in water and SNF (Table 1).

Before the statistical analysis of the obtained results, the input parameters were normalized to the unitless interval [−1,1]. This approach is customary in the experimental design modelling [41] and the analysis in the rest of the paper will be presented in the normalized terms (standardized covariates) to simplify obtained model equations which describe specific response variables.

Regression modelling (with linear effects and two-way interactions where applicable) was applied to derive an insight into which formulation and/or process parameters as well as their interactions are important to precisely estimate evaluated responses.

#### 3.1.1. Process Yield

Spray drying yield ranged between 13.9% and 68.6% (Table 1). The model obtained by the regression analysis exhibited a good fit (R-squared 0.67, RMSE 8.52 and Press RMSE 9.19) and can be presented by the following equation (Equation (8)):

Yield = 52.27 − 4.06 × HPMC + 6.6 × T_inlet-4.13 × FFR + 2.91 × HPMC × T_inlet + 6.4 × IT × FFR(8)

**Figure 1:**
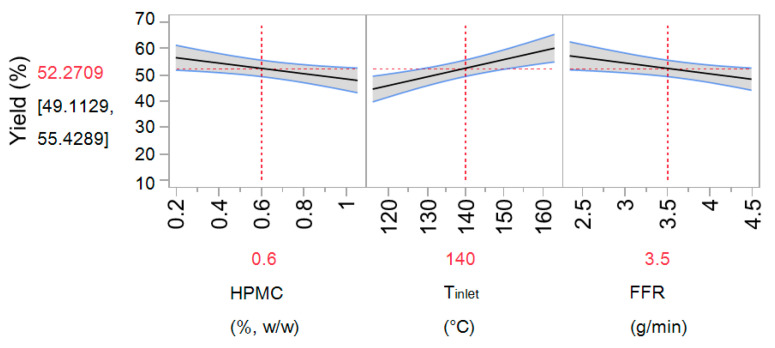
Prediction of yield in relation to hypromellose concentration (HPMC), inlet air temperature (*T*_inlet_) and feed flow rate (FFR). Values in brackets refer to 95% confidence interval.

The yield increased with the increase of inlet temperature and decrease of feed flow rate (Figure 1). These are commonly seen effects related to improved drying and decreased particle adhesiveness [42,43,44]. It was also noted that the process yield decreased with the increase of hypromellose concentration in the drying feed. Reduction of yield at higher hypromellose concentration may be related to the higher particle adherence on drying chamber walls resulting from the hydrophilic character of the polymer [45].

**Figure 2:**
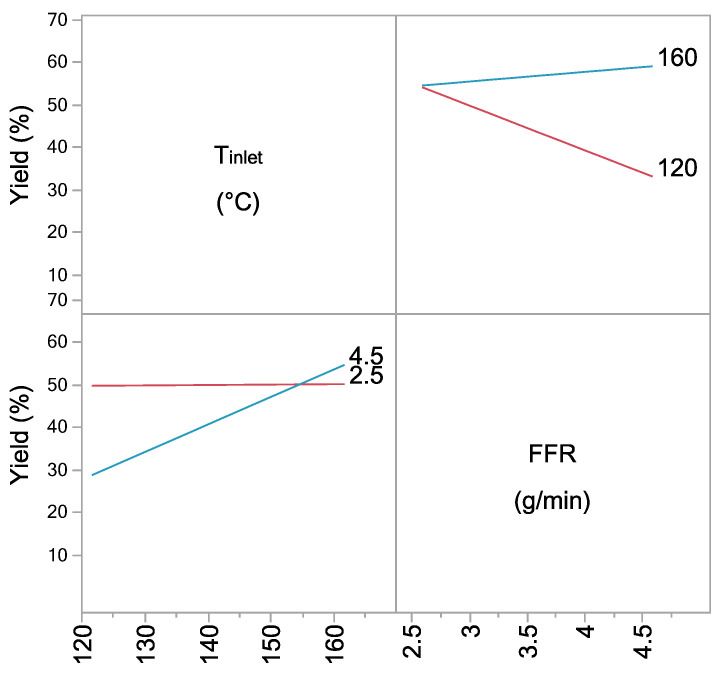
Interaction profiler illustrates the interaction effect between inlet air temperature (*T*_inlet_) and feed flow rate (FFR).

Interaction between inlet temperature and feed flow rate has shown a significant influence on the process yield (Figure 2). At *T*_inlet_ of 120 °C, the yield decreased with the increase in FFR, which was not observed at *T*_inlet_ of 160 °C due to better drying efficiency at higher temperature.

#### 3.1.2. Entrapment Efficiency and Drug Loading

All microspheres were characterised by high DSP entrapment efficiency (85.2 ± 4.8–101.9 ± 0.3%, Table 1) that was shown not to be correlated with inspected process and formulation parameters. Drug loading ranged between 1.4% and 33.5% being increased by increase of DSP concentration and decreased by hypromellose concentration increase in the feed solution. The model for drug content obtained by regression analysis exhibited a good fit (R-squared 1, RMSE 0.69 and Press RMSE 0.72) and is given by the following equation (Equation (9)):

(9) DL=12.87−5.3×HPMC+9.98×DSP−3.79×HPMC×DSP

#### 3.1.3. Particle Size

Results of microsphere size analysis for each experimental run are presented in Table 1. Values for *D*v10, *D*v50 and *D*v90 ranged between 1.4 ± 0.0–2.6 ± 0.3 µm, 2.2 ± 0.0–14.9 ± 3.4 µm and 3.7 ± 0.0–42.9 ± 0.6 µm, respectively. Values for *D*[4,3] ranged between 2.0 ± 1.2–18.5 ± 3.8 μm.

Models obtained by regression analysis exhibited a good fit for *D*v90 (R-squared 0.8, RMSE 5.83 and Press RMSE 6.99) and Dv10 (R-squared 0.68, RMSE 0.19 and Press RMSE 0.21) while *D*v50 (R-squared 0.46, RMSE 2.92 and Press RMSE 3.12) fit was not as good. Obtained models are presented by the following equations (Equations (10)–(12)):

(10) $$Dv10=1.73+0.24\times HPMC+0.11\times DSP+0.02\times T_{inlet}+0.07\times DSP\times T_{inlet}$$

(11) Dv50=4.46+2.22×HPMC+1.14×DSP+0.9×HPMC×DSP

(12) $$Dv90=13.2+8.67\times HPMC+2.26\times DSP-2.03\times T_{inlet}+2.08\times FFR-3.49\times HPMC\times T_{inlet}-2.33\times DSP\times FFR-3.32\times FFR\times T_{inlet}$$

Models for *D*v10, *D*v50 and *D*v90 reveal the highest impact of hypromellose concentration in the feed solution on the microsphere size distribution. Increase in hypromellose concentration resulted in larger microsphere sizes, which can be explained by higher viscosity of feed solution. Namely, for a given amount of energy available for atomisation process, increase in feed solution viscosity results in atomisation into larger droplets than in the case of less viscous feed, leading to larger size of dry particles [43]. Furthermore, increase in DSP concentration in the feed solution was shown to increase *D*v10, *D*v50 and *D*v90. This could be ascribed to increase in the solid content in the droplets to be dried. Except for the individual parameters, derived model for *D*v90 also included interactions between formulation and process parameters showing their interdependent effect on particle formation process [46].

Model obtained by regression analysis for *D*[4,3] (R-squared 0.62, RMSE 3.05 and Press RMSE 3.36) was given by the following equation (Equation (13)):

(13) D[4,3]=6.13+3.49×HPMC+1.25×DSP+0.3×FFR−1.24 DSP×FFR

The model confirmed hypromellose and DSP concentrations in the feed solution as parameters with the highest impact on microsphere size distribution.

#### 3.1.4. Residual Moisture Content

**Figure 3:**
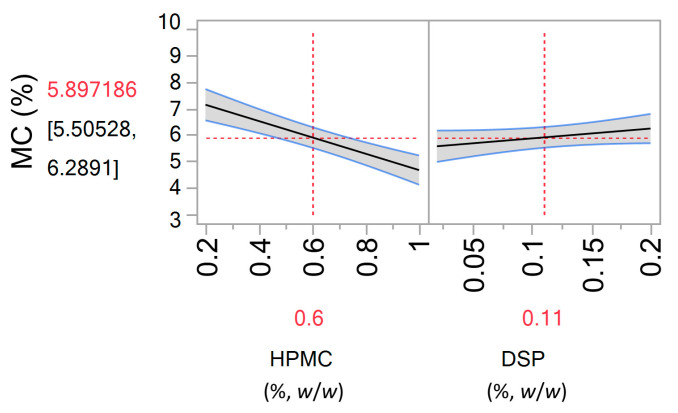
Prediction of moisture content (MC) in relation to hypromellose (HPMC) and DSP concentrations. Values in brackets refer to 95% confidence interval.

Residual moisture content (MC) in the microspheres prepared following designed experiments, ranged between 3.2 ± 0.8–9.1 ± 0.2%. The model obtained by regression analysis exhibited a relatively good fit (R-squared 0.57, RMSE 1.08 and Press RMSE 1.14; Figure 3) and is given by the following equation (Equation (14)):

(14) MC=5.9−1.23×HPMC+0.33×DSP

Residual moisture is shown to increase with the decrease in hypromellose and increase in DSP concentration in the feed solution. However, the values up to 10% of residual moisture content are acceptable for dry powder formulation based on hydrophilic polymers [47]. Decreasing hypromellose concentration in the feed solution led to microsphere matrix with higher pectin content as its concentration was kept constant in the feed solution. Thus, noted influence of hypromellose can be explained by higher hygroscopicity of pectin in relation to hypromellose, as confirmed by results on dynamic vapour sorption of polymers used reported in the literature [48]. The impact of DSP on residual moisture content in the microspheres can also be related to its hygroscopic nature [49].

#### 3.1.5. Swelling of the Microspheres

During the swelling process microspheres generally absorbed lower volumes of SNF (6.1 ± 1.6–23.5 ± 1.9 μL mg^−1^) when compared to purified water (6.7 ± 2.7–41.6 ± 3.4 μL mg^−1^). That can be explained by crosslinking of the pectin chains with calcium ions present in the SNF. Model obtained for swelling of microspheres in contact with water (*V*_water_) exhibited a good fit (R-squared 0.62, RMSE 4.96 and Press RMSE 5.18) and is given by the following equation (Equation (15)):

(15) $$V_{water}=26.67-5.05\times HPMC-4.17\times DSP$$

Volume of absorbed water decreased with the increase of hypromellose and DSP concentration in the feed solution. Increasing hypromellose and DSP concentration in the feed solution led to microsphere matrix with lower content of pectin. This is in agreement with previously shown better swelling properties of pectin in contact with water compared to hypromellose [22]. Higher content of drug entrapped reduced the amount of swellable polymers in the microsphere matrix.

Swelling of the microspheres in contact with SNF could not be adequately described by a regression model including inspected formulation and process parameters and their interactions. Such an observation leads to the conclusion that crosslinking of pectin with calcium ions present in SNF was the main factor governing the swelling behaviour.

Swelling of the microspheres in contact with nasal mucosa presents a prerequisite for exhibiting mucoadhesion properties and triggers release of the drug entrapped. Furthermore, microsphere swelling can cause dehydration of epithelial cells, leading to reversible widening of tight junctions and increase in the paracellular absorption for drugs [12]. However, excessive hydration of mucoadhesive polymers can result in slippery mucilage formation and reduction of adhesive strength [50]. Therefore, swelling behaviour moderated by means of pectin chains crosslinking with calcium ions is favourable for attaining prolonged residence time at nasal mucosa and adequate rate and extent of drug release.

#### 3.1.6. Selection of the Leading DSP-Loaded Microspheres

Models obtained through regression modelling revealed optimised process and formulation parameters in the production of DSP-loaded microspheres. Namely, spray drying of aqueous solution of DSP (0.2%, *w/w*), hypromellose (0.2%, *w/w*) and pectin (0.2%, *w/w*) at inlet temperature of 160 °C and feed flow rate of 2.5 g min^−1^ was identified as optimal for the production of moderately swelling DSP-loaded microspheres (sample 24; Table 1; further denoted as DSP-MS). Employed settings of inlet temperature (160 °C), feed flow rate (2.5 g min^−1^) and hypromellose concentration (0.2%, *w/w*) are linked to high process yield. In addition, selected hypromellose concentration was favourable as it resulted in particle size distribution shifted towards smaller particles. Namely, within the observed size range (Table 1), smaller particles are expected to exhibit stronger adhesion to coarse carrier particles as there is higher possibility for them to fit into irregularities of rough carrier surface which were previously shown to increase the attachment [21]. High DSP concentration (0.2%, *w/w*) in the spray drying feed is advantageous as it leads to the formation of microspheres with high drug content, leaving open the possibility of mixing the microspheres with inert carrier while not compromising the administration of therapeutic dose in the applicable amount of powder (up to 25 mg per nostril; [9]).

Observed residual moisture content in the selected microspheres was found not to influence the stability of incorporated drug, as drug loading within the microspheres stored in sealed containers at 4 °C for 12 months decreased for less than 2% (i.e., from 33.5 ± 0.4% to 31.9 ± 0.8%). Furthermore, microsphere size distribution upon 12 months of storage remained unchanged, as values of *D*v10, *D*v50, *D*v90 and *D*[4,3] (1.1 ± 0.0 µm, 3.2 ± 0.0 µm, 7.6 ± 0.0 µm and 4.1 ± 0.0 µm, respectively) were comparable to corresponding parameters determined immediately after microsphere preparation (1.5 ± 0.0 μm, 2.9 ± 0.0 μm, 8.2 ± 0.2 μm and 4.3 ± 0.1 µm, respectively; Table 1). Surface charge of microspheres was taken as another stability indicator: the values of zeta-potential measured immediately after microsphere preparation and upon 12 months of storage were −23.1 ± 1.4 mV and −22.0 ± 2.2 mV, respectively, confirming adequate microspheres’ stability profile. Moisture within the dry powder induces capillary forces at particle interfaces contributing to cohesive and adhesive forces in powder blends [51,52]. Thus, parameters of mixing of microspheres with inert carrier need to be matched with their properties in order to effectively disjoin microsphere agglomerates and produce homogenous adhesive powder blend [53].

#### 3.1.7. In vitro Release of DSP from Microspheres

**Figure 4:**
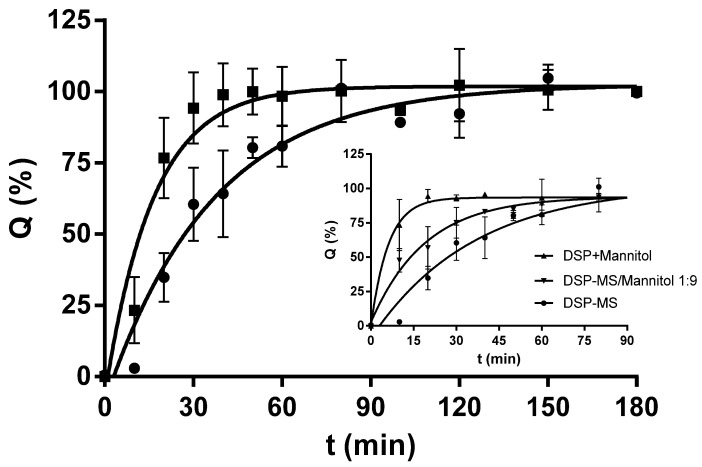
In vitro release profile of DSP from DSP-MS microspheres (circle) compared to dissolution of pure DSP powder (square). Graph insert: DSP in vitro release profile from DSP-MS microspheres blended with inert carrier (DSP-MS/Mannitol 1:9, *w/w*; reversed triangle) compared to dissolution of DSP from corresponding DSP/inert carrier blend (DSP + Mannitol; triangle). *Q* represents cumulative percentage of DSP released at time *t*. Data are expressed as the mean ± SD (*n* = 3).

The in vitro release profiles of DSP from microspheres in SNF were assessed under sink conditions using vertical Franz diffusion cell. The setup of the method allowed microspheres’ hydrating and gelling in conditions similar to ones at nasal mucosa [54]. It was observed that microspheres swelled in contact with SNF turning into gel. The formulation retained gel structure by the end of in vitro release study. Polymeric microspheres ensured prolonged DSP release in comparison to the pure drug powder (Figure 4). Two phases of drug release from microspheres could be recognised: rapid initial release (around 50% in 30 min) followed by a slower release (around 100% in 90 min) with ultimately resuming a plateau. Considering further formulation development that comprises blending of the selected microspheres with inert carrier, DSP release from microspheres in the presence of mannitol was also evaluated (Figure 4; graph insert). It was observed that DSP release rate from DSP-MS/mannitol blend (1:9, *w/w*) was increased in relation to that from DSP-MS alone. Similarly, shorter time-period was needed for complete dissolution of DSP from DSP/mannitol blend than from the pure DSP powder (Figure 4).

This effect might be related to osmotic activity of mannitol that increased powder wetting rate [9]. However, drug diffusion control by the swollen polymeric matrix of microspheres is evident from significantly slower DSP release from DSP-MS/mannitol blend in relation to DSP dissolution from corresponding DSP/mannitol blend. In contact with SNF, the inert carrier got dissolved and hydrophilic pectin and hypromellose in microspheres hydrated quickly forming a swollen hydrogel crosslinked with calcium ions which allowed adequate diffusion of drug molecules. The formulation retained gel structure by the end of in vitro release study. The observed release profile fit the needs for nasal DSP delivery showing the potential to initially provide high drug concentration at nasal mucosa favouring absorption and to ensure complete drug release within expected residence time of microspheres at nasal mucosa [9,22,55].

### 3.2. Properties of DSP-MS/Inert Carrier Blends

Powder blends were prepared by mixing of DSP-MS with lactose or mannitol at weight ratios of 1:9 and 1:19 using fraction of carrier particles in the size range from 45 to 63 μm, selected as optimal for nasal powder application [9,21].

**Figure 5:**
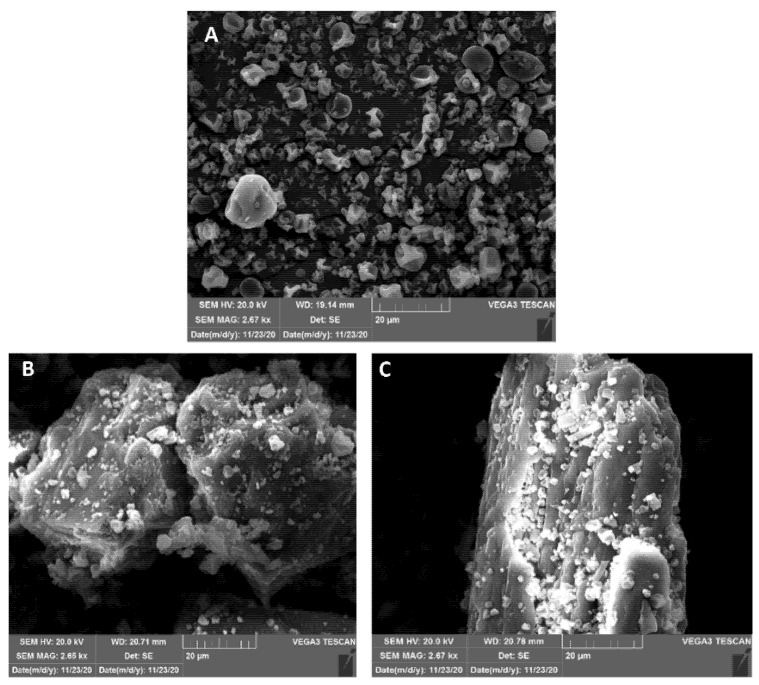
SEM micrographs of DSP-MS (**A**), DSP-MS/lactose blend (**B**) and DSP-MS/mannitol blend (**C**) with DSP-MS to inert carrier ratio 1:9, *w/w*.

SEM micrographs of DSP-MS and DSP-MS/inert carrier blends at ratio 1:9 (*w/w*) are shown in Figure 5. DSP-MS microspheres were spherical in shape with smooth and corrugated surface. A corrugated particle morphology was previously reported to reduce cohesiveness between the particles [56] which is beneficial for the production of homogenous DSP-MS/inert carrier blends. Carrier particles were characterised by irregular shape and rough surface. SEM micrographs of DSP-MS/inert carrier blends revealed DSP-MS adhered to mannitol or lactose particles, evenly covering their rough surface and fitting into the surface irregularities. It has previously been assumed that microparticles adhere better to rough carrier surfaces [21]. Such a phenomenon is beneficial for nasal powders since microspheres should stay attached to the carrier particles upon administration, co-depositing at the nasal mucosa. Rapid dissolution of carrier particles in nasal fluid liberates the microspheres enabling their intimate contact with nasal epithelium [21].

Homogeneity, flowability and sprayability of DSP-MS/inert carrier blends are presented in Table 2. Powder blends of DSP-MS with inert carrier (lactose or mannitol) at weight ratios of 1:9 and 1:19 were shown to be homogenous as mean values of percent amount of DSP to nominal dose in the taken powder blend samples (from bottom, medium and upper part of cuvettes) were in range of 100 ± 5.5%, with relative standard deviation (RSD) ≤ 5.5 [57]. The results obtained confirmed the effectiveness of mixing procedure employed and delivery of uniform dosage units. Apart from the mixing procedure, evolution of the blend state is influenced by the size and morphology of the carriers in a way that irregular and larger carriers favour deagglomeration of fine particles and their adhesion to open pores [58]. Namely, the carrier particles need to provide sufficient force to break up agglomerates of small particles during the mixing process [20].

Hausner ratios for DSP-MS, inert carriers and DSP-MS/inert carrier blends ranged between 1.14 ± 0.00 and 1.96 ± 0.18, as presented in Table 2. According to the Hausner ratio-based classification given in Ph. Eur. 10, the flowability of the investigated powders was denoted as good (for inert carrier) to very, very poor (for DSP-MS). Although Hausner ratio is not precisely predictive for non-freely flowable powder (e.g., spray-dried powder) behaviour, it certainly may show a trend in flow properties [59]. Thus, values of Hausner ratio obtained in this study clearly indicated that mixing of DSP-MS with inert carrier significantly improved powder flow properties (Table 2).

**Figure 6:**
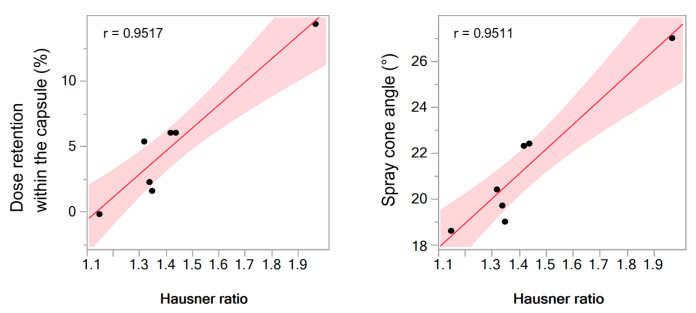
Correlation between dose percentage retained within the capsule and Hausner ratio (**left**; Pearson’s coefficient of 0.9517) and between spray cone angle and Hausner ratio (**right**; Pearson’s coefficient of 0.9511) established based on statistical analysis of data for DSP-MS, mannitol, lactose and DSP-MS blends with mannitol or lactose at weight ratios of 1:19 and 1:9.

Powder retention within the capsule upon device activation decreased with the decrease in Hausner ratio (Table 2) and served as a valuable and realistic indicator of powder flow behaviour. Statistical analysis performed on data for DSP-MS, mannitol, lactose and DSP-MS blends with mannitol or lactose at weight ratios of 1:19 and 1:9 revealed clear correlation of these variables described by Pearson’s coefficient of 0.9517 (Figure 6, left). Namely, percentage of the dose retained within the capsule for DSP-MS/inert carrier blends (1.7–6.3%; Table 2) was significantly lower than in case of DSP-MS alone (14.8%; Table 2). The same parameter indicated better flow properties of DSP-MS powder blends with mannitol in comparison to blends with lactose, reflecting delivery of almost 98% of the dose contained in the capsule. The difference in flow properties of DSP-MS powder blends with mannitol and lactose can be explained by the difference in the carriers’ particle morphology. Namely, mannitol particles were characterised by more pronounced surface roughness which might lead to stronger DSP-MS adherence [21]. No significant difference in percentage of dose retained within the capsule was noted between powder blends prepared at two different DSP-MS to inert carrier weight ratios (1:9 vs. 1:19).

**Figure 7:**
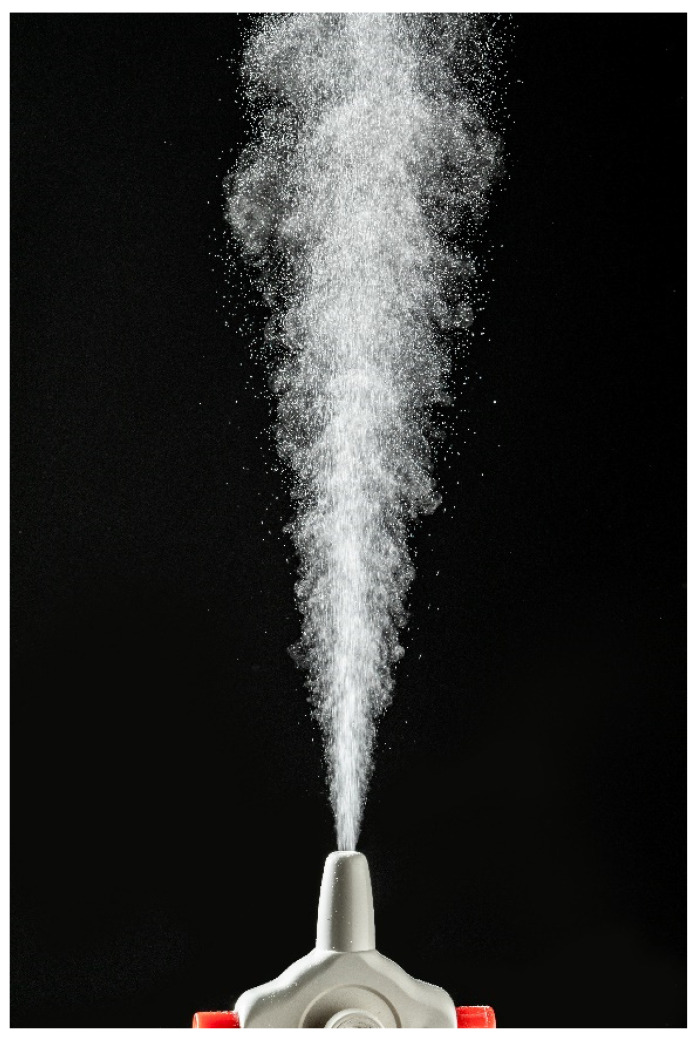
Spray cone of DSP-MS/Mannitol 1:9 upon aerosolisation with Miat^®^ insufflator.

The spray cone angle for all investigated powders ranged between 19.6 ± 1.0° and 26.5 ± 0.3° (Figure 7). Mixing of DSP-MS with inert carrier resulted in decrease in spray cone angle in relation to DSP-MS alone. Spray cone angle was shown to be related to powder flow properties as it decreased with the decrease in Hausner ratio. Statistical analysis performed on data for DSP-MS, mannitol, lactose and DSP-MS blends with mannitol or lactose at weight ratios of 1:19 and 1:9 revealed linear relationship between inspected variables, with Pearson’s coefficient of 0.9511 (Figure 6, right). The results obtained indicated smaller spray cone angle for DSP-MS blends with mannitol in relation to those with lactose which is advantageous as, generally, smaller spray cone angles increase the chance for drug delivery beyond the nasal valve [29].

### 3.3. *In Vitro* Mucoadhesion Studies

Mucociliary clearance has been recognised as one of the major limiting factors in nasal drug delivery since it reduces the time available for drug absorption to occur. Ciliated epithelial cells that provide mucus transport by the coordinated cilia beating are found in respiratory mucosa. However, mucociliary clearance also occurs in the olfactory region due to the presence of patches and islets of respiratory mucosa in the olfactory mucosa. Active clearance in olfactory region is also supported by Bowman’s glands secretion and gravitational forces reducing the time available for drug uptake to occur [60].

**Figure 8:**
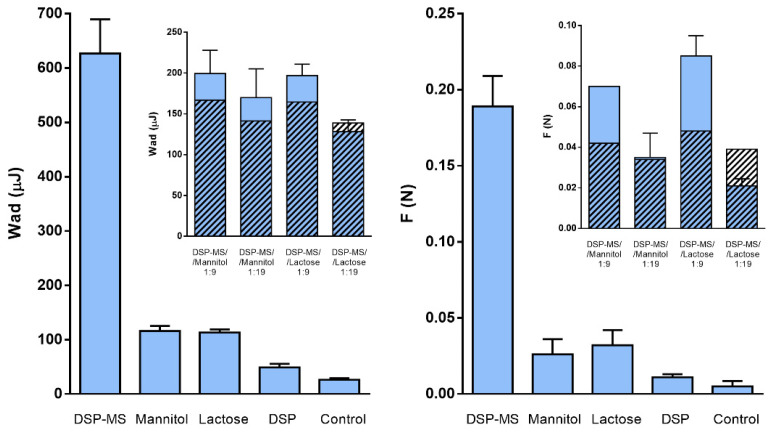
Work of adhesion (*W*_ad_; **left**) and maximum detachment force (*F*; **right**) of DSP-MS microspheres, inert carriers (mannitol and lactose) and pure drug powder compared to control (filter paper). Graph insert: Work of adhesion (*W*_ad_; **left**) and maximum detachment force (*F*; **right**) for DSP-MS/inert carrier blends at ratios 1:9 and 1:19 (*w/w*). For all graphs, bars coloured in blue represent measured values *W*_ad_ (**left**) and *F* (**right**) for 5 mg of each tested powder. Shaded bars represent theoretical values for *W*_ad_ (**left**) and *F* (**right**) calculated based on weight ratios and individual parameter values of powder blend constituents presented in the main graph. Data are expressed as the mean ± SD (*n* = 3).

Mucoadhesive drug delivery systems bear the potential to ensure improved drug therapeutic effect by prolonging its residence time at nasal mucosa and thus increasing its brain uptake [12,56]. Herein we investigated mucoadhesive properties of DSP-MS and DSP-MS/inert carrier powder blends using excised nasal porcine mucosa that was selected for this study due to its high similarity in histology and physiology with human nasal mucosa [61,62]. Mucoadhesion of powders was expressed as maximum detachment force and work of adhesion, as presented in Figure 8.

DSP-MS microspheres were characterised by prominent mucoadhesive properties, presenting 19 fold higher maximum detachment force and 12.8 fold higher work of adhesion in relation to pure drug powder.

Both pectin and hypromellose show mucoadhesive properties. Their adhesion to mucus layers is based on the interpenetration with mucin chains and hydrogen bond formation [63,64]. Hypromellose is a water-soluble electroneutral cellulose derivative that has a great number of hydroxyl groups available for interaction with the mucin glycoproteins [64]. Pectin bears carboxylic acid groups that are negatively charged at physiologic pH value, as indicated by overall negative zeta-potential of pectin/hypromellose DSP-MS microspheres (−23.1 ± 1.4 mV). The electrostatic repulsion between negatively charged pectin and mucin may contribute to uncoiling of polymer chains and facilitate their entanglement as well as hydrogen bond formation [63].

Mucoadhesive properties of DSP-MS microspheres are closely related to their swelling behaviour enabling interpenetration of polymer and mucin chains [65]. Excessive swelling that might lead to poor mucoadhesion has been avoided due to the presence of pectin in the polymer matrix that became crosslinked with the calcium ions present in SNF [22]. Measured *W*_ad_ and *F* for DSP-MS/inert carrier blends were significantly lower than that of DSP-MS alone. This was expected since microspheres presented only 5% or 10% of total quantity of examined powder blend. Therefore, in order to define the influence of inert carriers on DSP-MS mucoadhesive properties, we compared measured and theoretical values of *W*_ad_ and *F* for DSP-MS/inert carrier blends. Theoretical values were calculated based on weight ratios and individual *W*_ad_ or *F* values measured for powder blend constituents (Figure 8). Such an approach revealed that DSP-MS, when blended with inert carrier, retained or even enlarged their mucoadhesive properties in all inspected powder blends except for the blend with lactose at 1:19 weight ratio. The observed increase in mucoadhesive properties can be explained by the state of homogenous powder blends in which larger carrier particles induced deagglomeration of microspheres in the mixing procedure and promoted their adhesion to open pores.

When such a powder gets in contact with simulated nasal fluid the carrier particles rapidly dissolve [21], liberating microspheres that interact with the nasal mucosa more efficiently (i.e., with larger total surface area) than in case of DSP-MS alone that might be agglomerated. DSP-MS blend with lactose at 1:19 weight ratio (i.e., higher lactose content compared to 1:9 ratio) failed to retain or enlarge microsphere mucoadhesive properties, probably due to slower dissolution of lactose in relation to mannitol.

### 3.4. Biocompatibility of DSP-MS/Inert Carrier Blends

Biocompatibility of investigated powder delivery systems was evaluated in vitro using human airway epithelial Calu-3 cell line. The viability of cells exposed to DSP-MS/mannitol and DSP-MS/lactose powder blends at DSP concentration ranging from 0.45 mg mL^−1^ to 1.8 mg mL^−1^ and corresponding carrier concentration ranging from 12.7 mg mL^−1^ to 107.2 mg mL^−1^, was above 80% in relation to control. The results obtained suggested appropriate biocompatibility profile of all constituents of powder blends in concentration range tested.

Based on thorough characterisation of DSP-MS/inert carrier blends and recognised advantages of mannitol compared to lactose in the role of the carrier for DSP-MS microspheres, powder blends with mannitol were selected for further studies. Prior to in vitro permeability and nasal deposition studies, DSP-MS/mannitol blends were proven to be homogenous upon three months of storage in the sealed containers at 4 °C. Adequate powder blend homogeneity upon storage was confirmed by mean percentage ratio of experimentally determined and theoretical mass of DSP in the analysed samples of 99.0% and 99.4% and RSD value of 3.8% and 1.3% for DSP-MS/mannitol 1:9 and 1:19 blends, respectively.

### 3.5. *In Vitro* DSP Permeability Studies

DSP permeability studies were performed using immortalized Calu-3 cells grown at air-liquid interface as a model of the nasal epithelial barrier [66,67]. Such a model has been used in number of studies screening the potential of innovative nasal formulations intended for brain targeted drug delivery [68,69,70]. Air-liquid interface culturing of Calu-3 cells was previously shown to provide closer simulation of in vivo airway epithelia properties including monolayer ultrastructure, secretory function (i.e., mucus production) and barrier integrity, compared to liquid-liquid culturing [71].

In general, immortalized cell lines are characterized by easy maintenance, high throughput capacity as well as high reproducibility and genetic homogeneity [72,73], thus being convenient for the evaluation of different drug formulations in terms of drug permeation profile. The suitability of the Calu-3 cells for nasal epithelial barrier modelling has been proven by comprehensive permeability study on wide range of model drugs chosen from high to low permeability categories. The study revealed excellent correlation with the fully differentiated 3D human nasal epithelial model (MucilAir™) and good correlation with the human nasal epithelial cell line RPMI 2650 [72]. Finally, Calu-3 cell monolayer grown at air-liquid interface was recently shown to correctly predict the outcome of pharmacokinetic studies and indicate the (non)equivalence of different formulations for two first-generation intranasal corticosteroids with relatively high aqueous solubility [74].

In this study Calu-3 cell monolayer was used to investigate the influence of developed powder delivery platform on DSP permeability across the epithelial barrier. DSP-MS/mannitol powder blend at weight ratio of 1:19 was selected for this study in order to ensure both, hypertonic conditions in the donor compartment (expected in vivo) and sink conditions in the receiver compartment throughout the experiment. Table 3 presents all relevant test samples used to elucidate the key factors influencing DSP permeability across Calu-3 monolayer. DB solutions were also included in the study to prove the applicability of the barrier model employed.

Table 3 summarises *P*_app_ values for DSP and DB for all tested samples including their osmolalities and Calu-3 cell monolayer TEER drop during permeability studies.

*P*_app_ values for DB and DSP isoosmotic solutions in HBSS-Ca^2+^ across Calu-3 cell monolayer were determined to be 18.65 × 10^−6^ cm s^−1^ and 0.38 × 10^−6^ cm s^−1^, respectively, verifying the cell model capacity to differentiate between drugs with high and moderate permeability. Sibinovska and co-workers tested 22 model drugs with high, moderate and low permeability (defined according to the values of the orally absorbed fraction) in the air/liquid Calu-3 cell model. The *P*_app_ value for DB obtained in this study is in line with *P*_app_ values reported by Sibinovska et al. for highly permeable drugs (1.0 × 10^−5^ to 1.7 × 10^−5^ cm s^−1^). *P*_app_ value for DSP is within the *P*_app_ range the same group of authors obtained for drugs with moderate permeability (0.2 × 10^−6^ to 0.6 × 10^−6^ cm s^−1^) [72].

DB is a lipophilic drug that permeates biological barriers by transcellular pathway [31]. On the contrary, DSP is expected to permeate Calu-3 monolayer by paracellular route. This was evidently shown in our study as a 9-fold increase in *P*_app_ of DSP across Calu-3 cell monolayer was observed with the decrease in TEER that was induced by exposing the cell monolayer to hypoosmotic stress (Table 3; samples DSP and DSP-MS). Similar profound drop in TEER (to 29.2 ± 3.4% of initial TEER value) after apical cell exposure to hypotonic solution with osmolarity of 150 mOsmol was previously observed in case of Caco-2 cell monolayer [75]. At these conditions, a 12- and 8-fold increase in paracellular transport of hydrophilic model compounds (fluorescein-Na and fluorescein-isothiocyanate-labeled dextran, respectively) was reported. However, hypoosmotic stress induced no change in cell viability [75].

DSP/mannitol solution with osmolality of 470 mOsm kg^−1^ was characterised by DSP permeation across Calu-3 monolayer similar to that of isotonic DSP solution. DSP-MS/mannitol blend increased *P*_app_ value of DSP 1.7 fold in relation to corresponding DSP/mannitol solution suggesting the potential of developed microspheres to increase drug uptake by paracellular transport. The observed permeation enhancing effect of DSP-MS/mannitol powder blend was found to be statistically significant (*p* < 0.05) and, coupled with (i) prolonged drug release through swollen microsphere polymeric matrix and (ii) mucoadhesive properties (the benefit of which cannot be encompassed by static permeability model used), suggests that developed powder platform might increase overall DSP permeation across the olfactory epithelium in vivo.

As previously elaborated, DSP is a water-soluble sodium phosphate salt of dexamethasone that enables the administration of higher drug doses easily formulated in mucoadhesive powder delivery system with prolonged retention at the site of delivery. The approach suggested in this study has the potential to ensure double benefit, since based on the recent reports related to bioconversion of phosphate esters to more permeable forms by phosphatases present in nasal mucosa [17,19], it is reasonable to expect that the significant portion of the applied DSP dose will be absorbed in the form of dexamethasone base.

**Figure 9:**
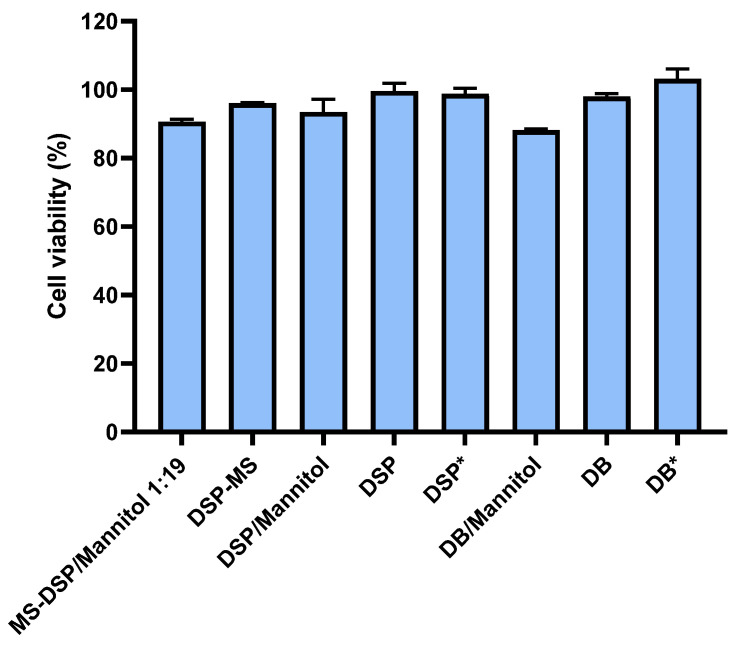
Viability of cells (MTT test) in Calu-3 cell monolayers used in permeability studies of DSP from DSP-MS suspensions and DSP solutions with and without mannitol. Corresponding DB solutions were also included in the study (see Table 3). Concentration of DSP, DB and mannitol (where applicable) in the test samples was equal to 0.9 mg mL^−1^, 0.075 mg mL^−1^ and 54.5 mg mL^−1^, respectively. Data are expressed as mean ± SD (*n* = 3). DSP * (*p* < 0.05); DB * (*p* < 0.05).

Viability of cells in Calu-3 cell monolayers used in permeability studies ranged between 88.2 ± 0.3 and 103.1 ± 2.9% in relation to cell monolayer exposed to HBSS-Ca^2+^ (Figure 9). Biocompatibility of the formulations was also confirmed by complete TEER recovery 22 h after permeability studies, showing that formulation effect on TEER was transient and reversible.

### 3.6. Nasal Deposition Profile of DSP Powder Formulations

Nasal deposition studies in vitro represent indispensable part of drug delivery system characterisation that complements the screening of its potential to ensure desired therapeutic effect. Owing to the efficiency in the treatment of neuroinflammatory processes, excessive first pass metabolism and systemic adverse reactions [76], dexamethasone represents the candidate for direct nose-to-brain delivery, which allows for reduction of the administered drug dose and systemic exposure. In case of dexamethasone, it might reduce the risk for systemic adverse reactions and decrease the extent of interaction with other drugs that are metabolized by CYP3A4, such as antiviral drug indinavir [77] investigated as a potential drug for the treatment of COVID-19 [78].

The direct nose-to-brain pathways start in olfactory and respiratory regions of the nasal cavity (via olfactory and trigeminal nerves, respectively). Olfactory pathway presents the most important route in this regard [8]. However, broader distribution to the respiratory mucosa innervated by the trigeminal nerve may also contribute to the brain targeted drug delivery [4,79].

**Figure 10:**
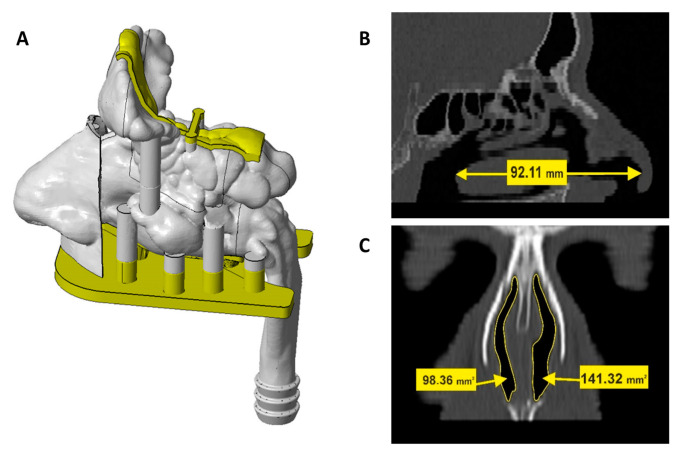
Schematic presentation of a 3D-printed nasal cavity model (**A**) and nasal geometry measurements including length of a nasal cavity (92.11 mm; (**B**) and smallest vertical cross-sectional areas (left: 98.36 mm^2^/right 141.32 mm^2^; (**C**).

In this work deposition studies were performed using a multi-sectional nasal cavity model (Figure 10A) developed using the CT scan of a patient with healthy nasal airways passages, with length of a nasal cavity (measured from the nostril to the end of the turbinates; [80]) of 92.1 mm (Figure 10B), and smallest vertical cross-sectional areas (valve region) of 141.3/98.4 mm^2^ (right/left, respectively; Figure 10C) fitting into the ‘normative’ mean (range) of 85 mm^2^ (20–160 cm^2^) [81]. The multi-sectional nasal cavity model enabled precise determination of drug deposited in the regions of interest, namely, olfactory region (superior turbinate with small portion of the middle turbinate and the corresponding segment of the nasal septum [82]) and respiratory region (the rest of turbinates innervated by trigeminal nerve and septum that are covered by respiratory epithelium).

Preliminary studies on nasal deposition of DSP-MS microspheres performed at administration angle of 60° and inspiratory air flow of 0 L min^−1^ confirmed inappropriate nasal DSP delivery by means of pure microsphere powder. This was related to poor DSP-MS flow properties leading to high powder retention within the capsule (Table 2), as well as to the loss of the sprayed powder at the entrance to the nasal cavity (visible during powder administration) and significant deposition outside the nasal cavity owing to small particle size. Blending of the microspheres with mannitol resulted in improved powder performance. As there was no crucial difference in the observed properties between DSP-MS/mannitol blends at 1:9 and 1:19 weight ratios (Table 2, Figure 8), nasal deposition was studied with the powder blend at higher drug dose (1:9, *w/w*).

The selected powder blend was shown to be homogenous upon 12 months of storage in sealed container at 4 °C, as mean percentage ratio of experimentally determined and theoretical mass of DSP in the analysed sample was 95.8%, with RSD value of 3.4%.

**Figure 11:**
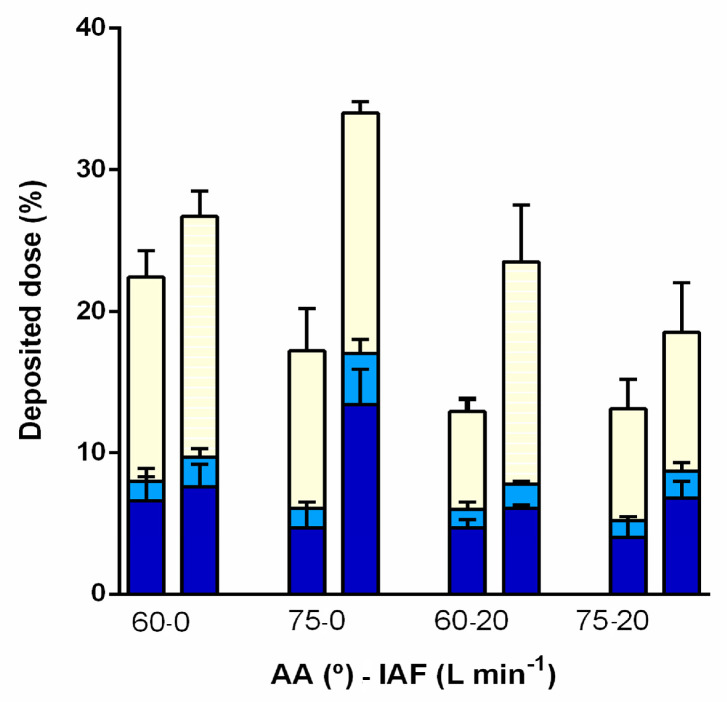
Nasal deposition of DSP-MS/Mannitol 1:9 in olfactory region (superior turbinate with a small portion of the middle turbinate (■) and corresponding segment of the nasal septum (■)) and the rest of turbinates innervated by trigeminal nerve (■) at various administration parameters in left and right half of 3D printed nasal cavity model (left and right bar, respectively). Administration parameters include angle of administration from horizontal plane (AA) and inspiratory airflow (IAF). All values are mean ± SD, *n* = 3.

The deposition in olfactory region (superior turbinate with a small portion of the middle turbinate and corresponding segment of the nasal septum) and the rest of turbinates innervated by trigeminal nerve upon administration of the powder blend at different angles from horizontal plane (60 and 75°) and inspiratory air flows (0 and 20 L min^−1^) are shown in Figure 11.

The fraction of the DSP dose deposited in the olfactory region ranged between 5.1 ± 0.9 and 17.0 ± 1.6% revealing the potential of developed powder platform for targeted olfactory delivery. For the comparison, available studies on nasal deposition of nebulised liquid formulation of albuterol sulphate as the model drug report less than 5% deposition in the olfactory region, for both, unilateral and bidirectional delivery [83], and even when different levels of nasal passage dilations were employed [84].

The highest fraction of the dose deposited in the olfactory region (17.0 ± 1.6%) and the highest total dose recovery (87.4 ± 2.1%) were obtained for powder blend administration at AA of 75° and breath hold (inspiratory airflow of 0 L min^−1^). Inspiratory airflow of 20 L min^−1^ was shown to reduce deposited DSP fraction in all regions of nasal cavity, resulting in low dose recovery (34.7 ± 1.5 to 50.3 ± 0.6%). Such observation is probably related to particle de-agglomeration enhanced by inspiratory airflow, leading to increased deposition outside the nasal cavity [21,22]. Favourable drug insufflation at breath hold is also advantageous from the patient’s point of view as it simplifies the mode of administration avoiding problems related to (i) coordination of breathing with powder actuation and (ii) producing adequately deep breath if drug should be administered under breathing conditions.

For all inspected administration parameters, fraction of the dose deposited in the turbinate region innervated by trigeminal nerve was relatively limited and ranged between 6.9 ± 0.9 and 17.0 ± 1.8% of the total dose. This fraction may contribute to direct nose-to-brain drug delivery via trigeminal pathway, however, it is also available for systemic absorption due to high vascularisation of nasal respiratory mucosa [69]. Therefore, moderate drug delivery to this region might be beneficial in the light of reducing the possibility for systemic drug effects. The fraction of the dose that is also available for systemic absorption is the one found on the septum outside the segment corresponding to olfactory region, and it ranged between 5.3 ± 0.6 to 12.6 ± 1.3%.

Fraction of the dose deposited in olfactory (and respiratory) region was higher when powder was administered to the right nostril of the 3D nasal cavity model, as right half was characterised by higher vertical cross-sectional area of valve region. This finding opens the possibility to adjust the administration strategy to individual patient nasal geometry when aiming olfactory region.

Conclusively, the fraction deposited in the olfactory region (up to 17.0%) can be considered as relatively large. Namely, direct nose-to-brain delivery implies avoidance of first pass effect as well as avoidance of dilution due to distribution and protein binding, therefore, the dose that needs to be delivered to the olfactory region to be absorbed by neuronal pathway may be as low as 0.01–1% of orally administered dose [85]. In this work, the highest fraction of the DSP dose (0.08 mg) delivered to the olfactory region (by single nasal administration of powder blend with DSP dose of 0.475 mg) represents 1% of dexamethasone oral daily dose (6 mg) recommended for the treatment of patients with severe and critical COVID-19 [86], clearly fitting to declared range.

## 4. Conclusions

QbD approach enabled rational design of spray-dried DSP-loaded polymeric microspheres. Optimised microspheres/mannitol powder blend showed favourable biopharmaceutical and sprayability properties considering the proposed route of administration. Nasal deposition studies revealed the potential of strategy employed for efficient delivery of DSP to the olfactory region of nasal cavity. The obtained results present a firm base for extending the study to an appropriate in vivo model needed for the final proof-of-concept.

## Figures and Tables

**Table 1:** Sample sequence from design of experiment and corresponding spray drying process yield, microspheres drug loading (DL), drug entrapment efficiency (EE), particle size distribution (*D*v10, *D*v50, *D*v90 and *D*[4,3]), residual moisture content (MC) and swelling properties expressed as volume of SNF (*V*_SNF_) and water (*V*_water_) absorbed per mg of microspheres in the swelling process.

	HPMC (%; *w/w*)	DSP (%; *w/w*)	*T*_inlet_ (°C)	FFR (g min^−1^)	Yield (%)	DL (%)	EE (%)	*D*v10 (µm)	*D*v50 (µm)	*D*v90 (µm)	*D*[4,3] (µm)	MC (%)	*V*_SNF_ (µL mg^−1^)	*V*_water_ (µL mg^−1^)
1	0.2	0.20	120	4.5	48.2	32.4 ± 0.5	97.2 ± 1.6	1.6 ± 0.0	2.7 ± 0.0	5.8 ± 0.1	3.3 ± 0.0	6.9 ± 0.6	13.6 ± 2.9	26.2 ± 4.2
2	1.0	0.20	160	4.5	52.4	14.6 ± 0.0	101.9 ± 0.3	1.9 ± 0.0	5.0 ± 0.0	17.7 ± 0.4	7.7 ± 0.1	4.7 ± 0.2	15.5 ± 1.4	22.4 ± 1.0
3	0.2	0.02	120	2.5	58.5	5.1 ± 1.6	97.9 ± 10.1	1.4 ± 0.0	2.2 ± 0.0	4.9 ± 0.0	2.0 ± 1.2	6.2 ± 0.4	11.7 ± 1.8	37.0 ± 2.0
4	0.6	0.11	140	3.5	63.5	11.7 ± 0.15	97.1 ± 1.2	1.6 ± 0.0	2.9 ± 0.0	8.1 ± 0.0	4.0 ± 0.0	5.0 ± 0.1	16.5 ± 2.6	20.7 ± 3.2
5	0.6	0.11	140	3.5	65.2	11.7 ± 0.15	97.1 ± 1.3	1.6 ± 0.0	3.0 ± 0.0	11.4 ± 0.4	5.0 ± 0.1	5.0 ± 0.1	16.1 ± 0.7	24.5 ± 0.4
6	0.2	0.02	120	4.5	50.7	4.3 ± 0.2	91.3 ± 3.2	1.4 ± 0.0	2.2 ± 0.0	4.4 ± 0.0	2.7 ± 0.0	5.7 ± 1.4	17.5 ± 2.2	39.2 ± 3.4
7	1.0	0.02	120	2.5	38.8	1.5 ± 0.0	91.8 ± 0.4	1.8 ± 0.0	3.3 ± 0.0	12.3 ± 0.1	5.4 ± 0.0	3.6 ± 0.5	16.3 ± 3.7	27.9 ± 2.0
8	1.0	0.20	120	4.5	25.2	13.2 ± 0.0	96.6 ± 5.8	1.8 ± 0.0	7.3 ± 0.9	36.6 ± 2.4	13.6 ± 0.3	3.2 ± 0.8	9.7 ± 1.4	16.0 ± 1.6
9	0.2	0.02	160	2.5	56.9	4.7 ± 0.0	98.6 ± 0.6	1.4 ± 0.0	2.2 ± 0.0	6.3 ± 0.0	3.6 ± 0.0	6.9 ± 0.5	14.8 ± 1.6	40.6 ± 4.0
10	0.2	0.20	160	4.5	68.6	31.7 ± 0.3	95.1 ± 1.0	1.6 ± 0.0	2.6 ± 0.0	5.1 ± 0.1	3.0 ± 0.0	6.6 ± 0.1	8.8 ± 1.0	24.8 ± 0.8
11	1.0	0.20	120	2.5	55.0	13.8 ± 0.3	99.4 ± 4.6	1.9 ± 0.0	4.1 ± 0.0	13.5 ± 0.3	6.9 ± 0.2	6.6 ± 0.0	15.0 ± 1.1	15.0 ± 3.7
12	0.2	0.02	160	4.5	53.2	4.6 ± 0.1	97.1 ± 1.6	1.4 ± 0.0	2.2 ± 0.0	4.4 ± 0.0	2.6 ± 0.0	7.7 ± 0.2	14.7 ± 2.9	41.6 ± 3.4
13	1.0	0.02	160	4.5	59.2	1.3 ± 0.0	85.2 ± 4.8	1.7 ± 0.0	3.6 ± 0.0	15.3 ± 0.3	5.4 ± 0.0	5.9 ± 0.0	14.3 ± 0.5	23.9 ± 1.0
14	0.2	0.20	160	4.5	66.6	31.7 ± 0.3	95.1 ± 1.0	1.7 ± 0.0	2.9 ± 0.0	6.0 ± 0.0	3.4 ± 0.0	6.6 ± 0.1	9.4 ± 1.0	21.9 ± 0.7
15	1.0	0.02	160	2.5	55.1	1.5 ± 0.0	97.6 ± 8.7	1.6 ± 0.0	3.1 ± 0.0	12.9 ± 0.0	5.4 ± 0.0	4.5 ± 1.9	23.5 ± 1.9	32.7 ± 0.5
16	1.0	0.02	120	2.5	65.9	1.5 ± 0.0	91.8 ± 0.6	2.0 ± 0.0	4.0 ± 0.0	10.8 ± 0.2	5.3 ± 0.1	3.6 ± 0.5	13.8 ± 2.7	30.2 ± 1.7
17	1.0	0.20	160	2.5	46.6	14.3 ± 0.1	101.6 ± 1.6	2.2 ± 0.1	13.7 ± 0.5	21.4 ± 0.9	13.3 ± 0.6	5.1 ± 0.1	19.5 ± 2.8	24.2 ± 2.2
18	0.2	0.20	120	2.5	56.7	32.9 ± 0.2	92.3 ± 8.9	1.5 ± 0.0	2.4 ± 0.0	4.8 ± 0.0	2.8 ± 0.0	9.1 ± 0.2	21.5 ± 1.4	35.7 ± 3.8
19	0.2	0.02	120	4.5	32.8	4.6 ± 0.4	96.7 ± 8.8	1.5 ± 0.0	2.2 ± 0.0	3.7 ± 0.0	2.4 ± 0.0	5.7 ± 1.4	14.7 ± 0.7	37.9 ± 3.0
20	0.2	0.02	160	4.5	51.7	4.6 ± 0.1	97.1 ± 1.6	1.5 ± 0.0	2.7 ± 0.0	7.1 ± 0.3	3.9 ± 0.2	7.7 ± 0.2	20.2 ± 2.9	32.1 ± 2.7
21	1.0	0.02	120	4.5	30.9	1.6 ± 0.1	100.4 ± 6.4	2.3 ± 0.0	12.7 ± 0.1	42.9 ± 0.6	18.0 ± 0.2	4.1 ± 0.4	9.9 ± 2.1	18.3 ± 0.4
22	1.0	0.20	120	4.5	13.9	13.2 ± 0.0	87.4 ± 7.2	2.0 ± 0.0	5.1 ± 0.2	34.0 ± 4.0	11.4 ± 0.3	3.2 ± 0.8	9.7 ± 1.4	16.0 ± 1.6
23	0.6	0.11	140	3.5	62.8	11.7 ± 0.2	97.1 ± 1.3	1.6 ± 0.0	2.8 ± 0.0	9.2 ± 0.4	4.4 ± 0.2	6.0 ± 0.2	6.1 ± 1.6	17.0 ± 3.3
24	0.2	0.20	160	2.5	57.0	33.5 ± 0.4	100.6 ± 1.3	1.5 ± 0.0	2.9 ± 0.0	8.2 ± 0.2	4.3 ± 0.1	8.7 ± 0.3	7.4 ± 2.1	23.6 ± 1.1
25	0.6	0.11	140	3.5	60.4	11.7 ± 0.2	97.1 ± 1.3	1.6 ± 0.0	2.7 ± 0.0	9.8 ± 0.3	4.7 ± 0.2	6.0 ± 0.2	9.4 ± 1.9	20.6 ± 0.9
26	0.2	0.20	120	4.5	42.8	32.4 ± 0.5	97.2 ± 1.6	1.7 ± 0.0	2.8 ± 0.0	5.3 ± 0.0	3.2 ± 0.0	6.9 ± 0.6	16.0 ± 0.6	23.8 ± 1.5
27	1.0	0.20	160	4.5	59.4	13.8 ± 0.2	96.6 ± 1.2	2.5 ± 0.0	6.5 ± 0.1	14.8 ± 0.6	7.7 ± 0.2	4.7 ± 0.2	15.5 ± 1.4	22.4 ± 1.0
28	1.0	0.02	120	4.5	25.9	1.6 ± 0.1	100.4 ± 6.4	1.7 ± 0.0	3.5 ± 0.0	36.0 ± 1.1	12.2 ± 0.4	4.1 ± 0.4	13.5 ± 0.7	22.5 ± 0.9
29	1.0	0.02	160	4.5	67.3	1.5 ± 0.1	93.9 ± 9.1	1.9 ± 0.0	3.9 ± 0.0	12.7 ± 0.1	5.8 ± 0.0	5.9 ± 0.0	14.3 ± 0.5	23.9 ± 1.0
30	0.2	0.20	120	2.5	57.5	32.9 ± 0.2	96.5 ± 3.0	1.5 ± 0.0	2.6 ± 0.0	6.3 ± 0.1	3.8 ± 0.1	9.1 ± 0.2	21.5 ± 1.4	35.7 ± 3.8
31	1.0	0.20	120	2.5	43.3	13.8 ± 0.3	96.4 ± 1.9	2.3 ± 0.0	14.9 ± 3.4	36.7 ± 7.8	18.5 ± 3.8	6.6 ± 0.0	13.4 ± 0.9	17.3 ± 0.9
32	1.0	0.20	160	2.5	55.6	14.3 ± 0.1	99.2 ± 1.9	2.6 ± 0.3	14.8 ± 2.0	26.7 ± 0.1	13.8 ± 0.0	5.1 ± 0.1	14.1 ± 0.5	19.4 ± 1.6

HPMC = hypromellose concentration in the spray drying solution; DSP = dexamethasone sodium phosphate concentration in the spray drying solution; *T*_inlet_ = inlet air temperature; FFR = feed flow rate. Values for the responses are mean ± SD, *n* = 3 (except for MC where *n* = 2).

**Table 2:** Properties of powder blends prepared with DSP-loaded microspheres (DSP-MS) and inert carrier (lactose or mannitol) at weight ratios of 1:9 and 1:19.

Powder Sample	Homogeneity		Flowability		Spray Properties
	D (%)	RSD (%)	Hausner Ratio	Powder Retention within Capsule (%)	CA (°)
DSP-MS	/	/	1.96 ± 0.18	14.8 ± 2.8	26.5 ± 0.3
DSP-MS/Lactose 1:9	100.2	4.6	1.43 ± 0.00	6.3 ± 1.7	22.0 ± 0.5
DSP-MS/Lactose 1:19	102.1	1.6	1.43 ± 0.10	6.0 ± 4.0	21.3 ± 0.4
DSP-MS/Mannitol 1:9	96.9	4.1	1.33 ± 0.00	1.7 ± 0.0	20.6 ± 0.2
DSP-MS/Mannitol 1:19	100.3	3.2	1.32 ± 0.07	1.7 ± 1.7	19.6 ± 1.0
Lactose	/	/	1.31 ± 0.10	5.5 ± 0.4	20.5 ± 0.6
Mannitol	/	/	1.14 ± 0.00	0.0 ± 0.0	18.7 ± 0.5

D = mean percentage ratio of experimentally determined and theoretical mass of DSP in the analysed samples (*n* = 6); RSD = relative standard deviation (*n* = 6); CA = spray cone angle. All values except for D and RSD are mean ± SD, *n* = 3.

**Table 3:** Permeation of DSP across Calu-3 monolayer from DSP-MS suspension and DSP solution in the presence and absence of mannitol. Corresponding DB solutions were also included in the study. Apparent permeability coefficients (*P*_app_) of DSP and DB were presented in relation to osmolality of the test sample and TEER drop of the Calu-3 cell monolayer during permeability study.

–	Medium	Osmolality (mOsm kg^−1^)	TEER % of Initial Value in the Period 30–120 min of Experiment	*P*_app_ (10^−6^ cm s^−1^)
DSP-MS/Mannitol 1:19	HBSS-Ca^2+^/water	613 ± 4	63 ± 2–94 ± 5	0.65 ± 0.12 ^‡^
DSP-MS	HBSS-Ca^2+^/water	160 ± 3	16 ± 2–22 ± 3	3.03 ± 0.01 ^‡^
DSP/Mannitol	HBSS-Ca^2+^/water	470 ± 1	53 ± 5–93 ± 5	0.39 ± 0.01
DSP	HBSS-Ca^2+^/water	162 ± 1	17 ± 1–20 ± 2	3.43 ± 0.62 ^‡^
DSP *	HBSS-Ca^2+^	304 ± 1	97 ± 4–103 ± 8	0.38 ± 0.06
DB/Mannitol	HBSS-Ca^2+^/water	460 ± 2	48 ± 6–73 ± 9	17.94 ± 1.61
DB	HBSS-Ca^2+^/water	161 ± 10	9 ± 1–13 ± 2	26.46 ± 2.94 ^†^
DB *	HBSS-Ca^2+^	298 ± 1	102 ± 4–104 ± 12	18.65 ± 1.92

Concentration of DSP, DB and mannitol (where applicable) in the test samples was equal to 0.9 mg mL^−1^, 0.075 mg mL^−1^ and 54.5 mg mL^−1^, respectively. Data are expressed as the mean ± SD (*n* = 3). ^‡^ statistically significant difference with respect to DSP/Mannitol and DSP * (*p* < 0.05). ^†^ statistically significant difference with respect to DB/Mannitol and DB * (*p* < 0.05).

## Data Availability

Not applicable.

## Supplementary Materials

The following are available online at https://www.mdpi.com/article/10.3390/pharmaceutics13060795/s1, Table S1. Parameters and their levels included in experimental design., Table S2. Validation parameters of HPLC method employed for DSP and DB analysis., Figure S1. Chromatogram of (**A**) standard dexamethasone sodium phosphate (DSP) and dexamethasone base (DB) solution (both at 2 μg mL^−1^), (**B**) sample of receptor medium taken during DSP permeability study form DSP powder formulation across Calu-3 cell monolayer, (**C**) eluate obtained by rinsing of the nasal cavity segment within nasal deposition studies of DSP powder formulation. All chromatograms were recorded at wavelength of 241 nm.
