# Supplementary material for: A Dry Powder Platform for Nose-to-Brain Delivery of Dexamethasone: Formulation Development and Nasal Deposition Studies
Source: Pharmaceutics. 2021 May 26;13(6):795. doi: 10.3390/pharmaceutics13060795 (PMC8229415; doi:10.3390/pharmaceutics13060795)
Supplement: Supplementary file 1 [file pharmaceutics-13-00795-s001.zip › pharmaceutics-1183808-supplementary.pdf]

pharmaceutics

Supplementary Materials: A Dry Powder Platform for Nose-To-Brain Delivery of Dexamethasone: Formulation Development and Nasal Deposition Studies

Laura Nižić Nodilo, Ivo Ugrina, Drago Špoljarić, Daniela Amidžić Klarić, Cvijeta Jakobušić Brala, Mirna Perkušić, Ivan Pepić, Jasmina Lovrić, Vesna Saršon, Maša Safundžić Kučuk, Dijana Zadravec, Livije Kalogjera and Anita Hafner

Table S1. Parameters and their levels included in experimental design.

Formulation Parameters	Low (-1)	Medium (0)	High (+1)
DSP concentration, DSP (%; *w/w*)	0.02	0.11	0.2
Hypromellose concentration, HPMC (%; *w/w*)	0.2	0.6	1.0
**Process parameters**	**Low (-1)**	**Medium (0)**	**High (+1)**
Inlet air temperature, T_{inlet} (°C)	120	140	160
Feed flow rate, FFR (g min^{-1})	2.5	3.5	4.5

Table S2. Validation parameters of HPLC method employed for DSP and DB analysis.

	**Range of linearity**					
DSP	1–15 µg/mL					
DB	1–15 µg/mL					
	**Linearity**					
	regression equation*			correlation coefficient		
DSP	y = 35033x – 18226			0.998		
DB	y = 52032x - 12208			0.999		
	**Precision Data**					
	repeatability (RSD %)			intermediate precision (RSD %)		
	low	medium	high	low	medium	high
	2 µg/mL	7.5 µg/mL	15 µg/mL	2 µg/mL	7.5 µg/mL	15 µg/mL
DSP	1.2	1.3	1.2	1.5	0.9	0.8
DB	1.3	1.6	1.0	1.2	1.4	1.1
	**Accuracy Data**					
	accuracy (recovery, mean (%) ± RSD, *n* = 3)					
	low (2 µg/mL)		medium (7.5 µg/mL)		high (15 µg/mL)	
DSP	103.0 ± 0.6		100.0 ± 0.5		99.8 ± 0.2	
DB	99.5 ± 1.3		96.2 ± 1.2		100.4 ± 0.8	
	**Sensitivity**					
	LOD (µg/mL)			LOQ (µg/mL)		
DSP	0.65			1.98		
DB	0.63			1.91		

*x—concentration (µg/mL); y—Area under Curve (µV s); All measurements were performed in triplicate.

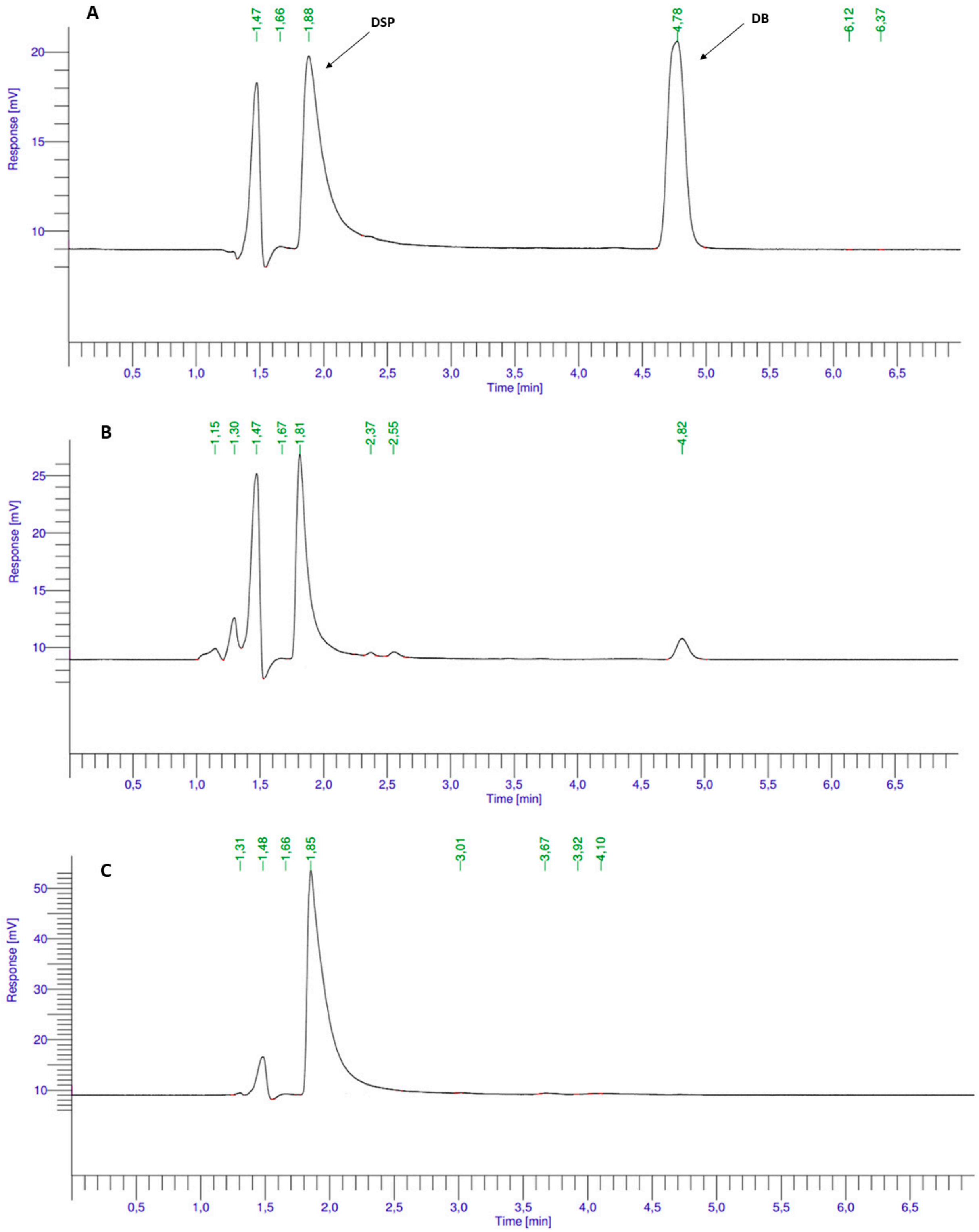

Figure S1. Chromatogram of (**A**) standard dexamethasone sodium phosphate (DSP) and dexamethasone base (DB) solution (both at 2 µg mL^{-1}), (**B**) sample of receptor medium taken during DSP permeability study form DSP powder formulation across Calu-3 cell monolayer, (**C**) eluate obtained by rinsing of the nasal cavity segment within nasal deposition studies of DSP powder formulation. All chromatograms were recorded at wavelength of 241 nm.
